# A Review and Experimental Analysis of Supervised Learning Systems and Methods for Protein–Protein Interaction Detection

**DOI:** 10.3390/ijms27094094

**Published:** 2026-05-02

**Authors:** Kamal Taha

**Affiliations:** Department of Computer Science, Khalifa University, Abu Dhabi P.O. Box 127788, United Arab Emirates; kamal.taha@ku.ac.ae

**Keywords:** protein–protein interaction, PPI, supervised learning, machine learning models, ELM, CNN, GNN, SVM, KNN

## Abstract

The exponential growth of genomic and proteomic data has made computational protein–protein interaction (PPI) prediction indispensable, driving the need for a comprehensive and method-aware evaluation of supervised learning approaches. PPIs are fundamental to understanding cellular processes and disease mechanisms, yet experimental identification remains slow, costly, and difficult to scale. This survey systematically investigates ten supervised learning models—Extreme Learning Machine (ELM), Convolutional Neural Networks (CNNs), Graph Neural Networks (GNNs), Deep Neural Networks (DNNs), Naïve Bayes, Probabilistic Decision Tree, Support Vector Machine (SVM), Least Squares SVM (LS-SVM), K-Nearest Neighbor (KNN), and Weighted K-Nearest Neighbor (WKNN)—through a tri-layered framework that integrates *Comparative Quantitative Analysis*, *Comparative Observational Analysis*, and *Experimental Evaluations*. Beyond conventional accuracy summaries, this work provides *critical commentary tied to real-world use*, analyzing where techniques succeed or fail in practice—for instance, when instance-based methods bottleneck during inference, when kernel choices influence SVM variance, or when deep architectures trade accuracy for computational cost. The survey also offers concrete deployment guidance, such as calibration insights for WKNN versus KNN under varying feature noise or dataset curation quality, delivering operational perspectives that typical surveys omit. Comparative Quantitative Analysis consolidates metrics such as accuracy, F1-score, and computational time from the existing literature, while Comparative Observational Analysis evaluates interpretability, scalability, dataset suitability, and efficiency. Complementing these, *Experimental Evaluations* conducted by the authors empirically validate model performance on benchmark datasets. Together, these layers provide a unified and evidence-backed perspective on algorithmic strengths, weaknesses, and practical applicability. Findings show that GNNs and DNNs achieve the highest predictive accuracy due to their ability to capture structural and topological relationships, whereas ELM and Naïve Bayes offer superior efficiency. SVM and LS-SVM maintain robust stability under noisy conditions, and CNNs are well-suited for sequence-based prediction tasks. By combining empirical validation, critical insights, and deployment-focused recommendations, this survey delivers decision-grade guidance that bridges theoretical understanding with real-world implementation, thus clarifying the trade-offs among accuracy, efficiency, and scalability in PPI detection research.

## 1. Introduction

Proteins, composed of amino acids, are fundamental to biological functions, such as DNA replication and molecular transport within organisms. They are essential to protein–protein interactions (PPIs), which drive numerous biological processes. Disruptions in these interactions can lead to diseases such as cervical leukemia and tuberculosis. PPIs represent specific interactions between proteins, influenced by forces like electrostatic interactions, thus highlighting the intricate molecular complexity within cells. These interactions are crucial for forming large molecular assemblies and enzyme complexes [1]. Also, PPIs are integral to various cellular functions, thus offering valuable insights into cellular mechanics and potential therapeutic applications. From cellular communication to metabolic regulation, PPIs are central to maintaining biological systems [2].

Advancements in studying protein interactions have led to the development of PPI networks, which exhibit scale-free properties [3,4,5,6,7]. This research area has garnered attention in high-impact journals such as *Nature* [8], *Science* [9,10], and the *Proceedings of the National Academy of Sciences* [11,12]. As the volume of PPI data grows, efficient management has become essential, driving the development of specialized tools and databases [8,13]. PPI data is collected through high-throughput screening techniques [8,13], computational studies [14,15,16,17], and literature mining approaches [18,19]. The integration of these diverse data sources has propelled ongoing research advancements. Repositories like BIND [20], DIP [21], IntAct [22], and HPRD [23] have been established to systematically organize and manage PPI data. Open-source software designed for these databases supports the extensive selection and processing of PPI information, while continuous improvements in detection methods are expanding the boundaries of PPI research.

Traditional experimental methods, such as Yeast Two-Hybrid screening, have significantly contributed to PPI discovery but are limited by scalability, high costs, and labor-intensive requirements. With the growing availability of genomic and proteomic data, computational approaches have become indispensable for predicting PPIs, particularly in advancing drug discovery and personalized medicine. Machine learning (ML) has shown remarkable potential in identifying patterns within complex biological datasets [7,24,25]. By integrating diverse biological data, ML techniques provide insights beyond the reach of traditional methods. These advancements hold the promise of revolutionizing our understanding of biological systems and driving innovations in biomedical research. However, several challenges remain in developing robust ML models, including addressing imbalanced datasets, selecting relevant features, optimizing hyperparameters, and validating predictions. Overcoming these challenges requires a multidisciplinary approach that combines computational expertise, biological knowledge, and understanding of the data.

This paper aims to address the growing importance of PPI prediction due to its critical role in understanding biological systems and advancing therapeutic development. Traditional experimental methods for PPI discovery are limited by scalability, high costs, and labor-intensive requirements. The increasing availability of genomic and proteomic data has driven a shift toward computational approaches, especially those utilizing statistical supervised learning algorithms, to overcome these limitations. By offering a systematic review and experimental evaluation of these approaches, this work fills a significant gap in the literature by enabling researchers to identify optimal methods for specific PPI tasks while advancing the understanding of ML applications in bioinformatics. This work follows a structured review methodology guided by systematic search principles, but it does not claim full compliance with formal systematic review protocols such as PRISMA.

## 2. Motivation and Key Contributions

### 2.1. Motivation

PPIs are vital to cellular functions, yet experimental identification is costly and slow. With the surge of genomic and proteomic data, computational prediction has become essential but challenging. This survey systematically evaluates supervised learning methods for PPI detection through both empirical and experimental analyses.

A central motivation of this survey is to give readers decision-grade guidance grounded in both the literature and our own experiments. To that end, we adopt a three-layer framework—Comparative Quantitative Analysis, Comparative Observational Analysis, and Experimental Evaluations—that jointly addresses accuracy, efficiency, scalability, and interpretability, turning disparate results into coherent, actionable evidence.

### 2.2. Why This Survey—What Is Different and Better

**Figure 1:**
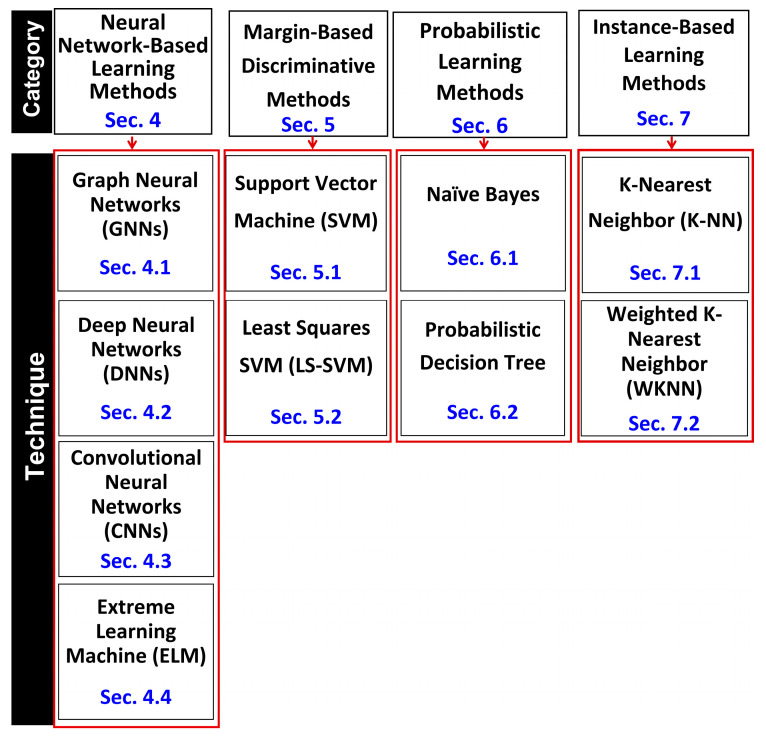
Illustration of our methodology-based taxonomy, which organizes algorithms for PPI detection into detailed classes using a hierarchical structure comprising categories and techniques. For each level—category and technique—the figure specifies the corresponding section number in the manuscript where it is discussed.

- Triangulated evidence, not summaries: Unlike prior reviews that stop at collating reported metrics, we triangulate literature-derived aggregates with observational (qualitative, deployment-oriented) comparisons and with our own experiments on benchmark datasets. This layered design lets readers reconcile “headline” metrics with operational constraints (compute budget, data regime, interpretability needs).
- Critical commentary tied to real-world use: Beyond accuracy tables, we analyze where techniques succeed or fail in practice—for example, noting when instance-based methods bottleneck at inference, when kernel choices drive SVM variance, and when deep stacks trade performance for compute. We also provide concrete deployment guidance (e.g., WKNN vs. KNN calibration and when to favor each under feature noise or curation quality), which typical surveys omit.
- A methods-first taxonomy with pointers to sections (Figure 1): We organize the space by methodology (from ELM/CNN/GNN/DNN to probabilistic and margin-based learners) and explicitly link taxonomy nodes to manuscript sections, improving navigability for readers who need technique-specific depth quickly.
- Clear, prescriptive takeaways: We convert the comparative analyses into strategic recommendations—what to use for high-accuracy scenarios, real-time triage, quick prototyping, or when interpretability dominates—so researchers can immediately map method to constraint.

### 2.3. Key Contributions

- Three-Layered Evaluation (Empirical + Experimental): We integrate (a) *Comparative Quantitative* results (averaged accuracy, F1, and time across studies), (b) *Comparative Observational* assessments (scalability, interpretability, dataset fit, efficiency), and (c) *our Experimental Evaluations*, offering a balanced, multi-angle evidence base that goes beyond single-view meta-analysis.
- Critical Commentary and Insights for Supervised PPI Methods: For each family—ELM, CNNs, GNNs, DNNs, Naïve Bayes, Probabilistic Decision Trees, SVM/LS-SVM, KNN/WKNN—we articulate *why* and *when* performance differs (e.g., CNNs capture sequence motifs but miss long-range structure; GNNs exploit topology and interfaces; ELMs deliver ultra-fast training but can trade off specificity), anchoring commentary in both literature trends and experimental behavior.
- Deployment-Aware Guidance: We translate findings into practical rules of thumb: prefer GNNs/DNNs for maximal accuracy on large or structure-rich data; use ELM for large-scale, real-time screening; apply Naïve Bayes for rapid prototyping; treat KNN/WKNN cautiously at scale due to inference cost, with calibration tips when local distances are reliable.
- Evidence-Backed Rankings with Nuanced Trade-offs: Our synthesis highlights GNNs and DNNs as accuracy leaders, while ELM and Naïve Bayes dominate efficiency; we also surface cases where classical variants (e.g., LS-SVM) stabilize performance under noise. This enables principled model selection rather than one-size-fits-all claims.
- What Readers Gain vs. Existing Surveys: Readers receive (a) *triangulated* results (not just curated citations), (b) *qualitative, operations-focused analysis* aligned to real datasets and resource envelopes, (c) *section-indexed taxonomy* for fast lookup, and (d) *prescriptive recommendations* by scenario; these are elements that are typically missing or treated separately in prior work.
- Bridging Theory and Practice: By combining theoretical underpinnings, literature-level aggregation, and hands-on experiments, the survey clarifies accuracy–efficiency–interpretability trade-offs and accelerates method-to-use-case mapping in biomedical pipelines.
- End-user takeaway: For sequence-only settings with tight compute, start with CNNs (or ELM for ultra-fast triage). When structure or topology is available, or when interfaces matter, favor GNNs. Where peak accuracy is paramount, and where resources permit, use DNNs—ideally paired with graph reasoning. These prescriptions emerge from the combined empirical and experimental evidence synthesized in this work.

## 3. Review Methodology and a Standardized Evaluation Scale (Scale 1–4)

***Eligibility Criteria:*** Studies were included if they: (1) were published between 2005 and 2025; (2) were written in English; (3) proposed or evaluated supervised learning approaches for PPI prediction; (4) reported empirical results using benchmark datasets.

***Exclusion Criteria:*** Studies were excluded if they: (1) focused purely on experimental wet-lab validation without computational modeling; (2) addressed PPI extraction from text without biological interaction modeling; (3) proposed unsupervised clustering without supervised classification; (4) lacked empirical evaluation.

***Search Strategy:*** The literature search covered: (1) IEEE Xplore; (2) ACM Digital Library; (2) Web of Science; (3) Scopus; (4) PubMed; (5) ScienceDirect; (6) SpringerLink

***Representative search query:*** (“protein–protein interaction” OR “PPI prediction”) AND (“supervised learning” OR “SVM” OR “CNN” OR “GNN” OR “ELM” OR “Naïve Bayes” OR “KNN”.

***Description of the screening process.***

- The total number of papers initially retrieved from the specified databases is 146.
- The number of papers after removing duplicates is 128.
- The number of papers excluded based on title/abstract/content screening is 58.
- The final number of papers included in the survey is: (1) 70 papers, whose proposed methods are discussed; (2) 30 papers that are only referenced.

This survey adopts a unified four-point Standardized Evaluation Scale (1–4). Each research article and explanation technique is evaluated using the same set of clearly defined metrics, ensuring objectivity, reproducibility, and direct comparability across methodological families. Each column in the evaluation tables corresponds to a specific metric, and each score (1–4) is grounded in explicitly defined qualitative criteria, allowing readers to transparently interpret and compare results across techniques. Table 1 shows the scale.

***Clarification on how the scores were assigned:***

- *Accuracy scores* were assigned based on reported quantitative metrics (e.g., AUROC, F1-score, MCC) and robustness across datasets.
- *Scalability scores* were determined using dataset size, computational complexity, and evidence of large-scale applicability.
- *Efficiency scores* were based on reported training cost, runtime behavior, and model complexity.
- *Interpretability scores* were assigned through qualitative assessment of model transparency, availability of explanation mechanisms, and biological interpretability.

***The scoring combines the following:***

- Reported empirical results from the literature.
- Computational characteristics of each method.
- Observational (qualitative) analysis.

***Below are the types of features used across all ten supervised learning models discussed in the survey:***

- Sequence-based features (e.g., amino acid composition, k-mer frequencies, evolutionary profiles);
- Physicochemical properties (e.g., hydrophobicity, charge, polarity);
- Structural features (e.g., 3D conformations, residue contact maps);
- Network/topological features (e.g., node degree, graph connectivity in PPI networks);
- Embedding-based features (e.g., protein language model embeddings, learned representations).

## 4. Neural Network-Based Learning Category

### 4.1. Graph Neural Networks (GNNs) Technique

#### 4.1.1. Components and Rationale of GNNs for PPI Prediction

**Figure 2:**
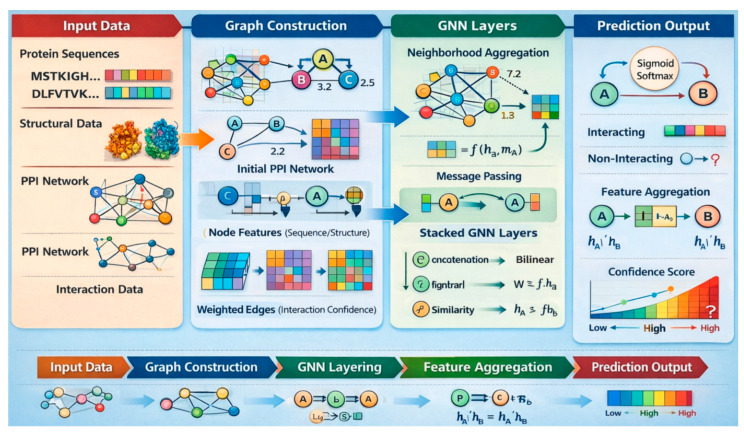
GNNs for PPI Prediction. Protein data and known interaction information are first encoded as node features, while experimentally validated or predicted interactions form weighted edges in the initial PPI graph. During graph construction, each protein is represented as a node $v_{i}$ with feature vector $h_{i}^{\left(0\right)}$, and edges $e_{ij}$ may incorporate confidence scores or similarity weights. The GNN layers perform iterative neighborhood aggregation through message passing, where node embeddings are updated as $h_{i}^{\left(l+1\right)}=\sigma (W^{\left(l\right)}\cdot \mathrm{AGG}\{h_{j}^{\left(l\right)}:j\in N(i)\})$, enabling integration of local and higher-order topological information. Stacked GNN layers capture multi-hop dependencies within the PPI network, preserving structural context and relational patterns. The learned protein embeddings are then combined using operations such as concatenation, bilinear transformation, or similarity scoring to form pairwise interaction representations. Finally, a sigmoid or softmax layer produces the interaction prediction (interacting vs. non-interacting) along with a confidence score, trained using cross-entropy loss and optimized via backpropagation.

GNNs are composed of key components, including node feature initialization, message passing, aggregation, and update functions. In the context of PPI prediction, proteins or amino acids are represented as nodes, and their structural or functional relationships are modeled as edges in a graph. During message passing, each node gathers information from its neighbors, which is then aggregated and used to update its own representation. This process is repeated across multiple layers, allowing the model to capture both local and global structural dependencies within the protein interaction network. Figure 2 depicts the GNN structure. GNNs are well-suited for PPI prediction because protein structures and interaction networks are inherently graph-based. They excel at capturing complex topological relationships and dependencies between proteins that traditional models may overlook. By learning from both node features (e.g., sequence embeddings) and network topology, GNNs can effectively identify latent interaction patterns.

#### 4.1.2. Mathematical Formulation of GNNs

GNNs operate on a graph *G* = (*V*, *E*), where *V* is the set of nodes (e.g., proteins or amino acids) and *E* ⊆ *V* × *V* represents the set of edges (e.g., physical or functional interactions). Each node *v* ∈ *V* is associated with an initial feature vector $h_{v}^{\left(0\right)}\in {\mathbb{R}}^{d}$, typically derived from protein sequence embeddings, physicochemical attributes, or structural data. The core mechanism of GNNs is *message passing*, where the feature of each node is iteratively updated by aggregating information from its neighbors. For each layer *l* ∈ {1, 2,…, *L*}, the node embedding $h_{v}^{\left(0\right)}$ is updated as shown in Equation (1):

(1) $$h_{v}^{\left(l\right)}=\sigma \left(W^{\left(l\right)}\cdot {\mathrm{AGGREGATE}}^{\left(l\right)}\left(\left\{h_{u}^{\left(l-1\right)}|u\in N(v)\right\}\right)+b^{\left(l\right)}\right)$$

where *N*(*v*) is the set of neighboring nodes of *v*, AGGREGATE^(l)^ is a permutation-invariant function (e.g., sum, mean, or max), W^(*l*)^ and b^(l)^ are learnable weight matrix and bias vector, and σ is a non-linear activation function (e.g., ReLU). The final node representation $h_{v}^{\left(l\right)}$ encodes information from its local neighborhood up to *L* hops away. For *PPI prediction*, a pairwise scoring function is applied to node embeddings to infer whether two proteins interact, as shown in Equation (2):

(2) $${\overset{⌢}{y}}_{uv}=f\left(h_{u}^{\left(L\right)},h_{v}^{\left(L\right)}\right)=\sigma \left(h_{u}^{\left(L\right)}{\mathrm{Mh}}_{v}^{\left(L\right)}\right)$$

where ${\overset{⌢}{y}}_{uv}$ is the predicted interaction probability between proteins *u* and *v*, M is a learnable similarity or interaction matrix, and σ ensures output lies in the range [0, 1].

#### 4.1.3. Featuring and Evaluating Research Papers That Have Utilized GNNs for PPI Detection

Zhang et al. [26] introduced MFC-PPI, which predicts multi-label PPIs by fusing multimodal protein features and using contrastive learning to strengthen representations. Arteaga et al. [27] introduced GSMFormer-PPI, which is a multimodal transformer that integrates protein molecular surface descriptors, 3D structural graphs, and residue-level language-model embeddings for PPI prediction. Yao et al. [28] use generative + contrastive self-supervised learning over PPI networks to identify virulence factors as a node-classification problem. Zhang et al. [29] introduced DSSGNN-PPI, which improves multi-label PPI type prediction by learning both local and global graph representations. Bi et al. [30] introduced SemanGraphPPI, which predicts PPIs by injecting semantic knowledge from a GO knowledge graph.

Park et al. [31] introduced XGPN, which is an explainable graph propagational network proposed for classifying mild cognitive impairment subtypes using graph propagation to capture global effects. Daou et al. [32] introduced MComplex, which identifies protein complexes from temporal/dynamic PPI networks by integrating time-series gene expression with interaction data. Wang et al. [33] performed full-atom PPI prediction using an atomic equivariant attention network that explicitly models residue/atom geometry for interaction inference. Li et al. [34] proposed a temporal protein complex identification method using a dynamic heterogeneous protein information network representation. Chiaranaipanich and Achakulvisut [35] introduced PPIM-Struct, which predicts PPI modulator activity using PPI structure-based representations.

Chen et al. [36] introduced MEGAE, which uses multiview heterogeneous graph autoencoders with random masking for PPI prediction and PPI-site inference. Yang et al. [37] introduced PepGFD, which predicts protein–peptide binding residues. Zhang et al. [38] introduced GDTGO, which is a graph-based predictor for PPI prediction using iterative optimization and representation learning. Mao et al. [39] introduced LG-MDVGA, which performs link prediction for lncRNA–protein interactions using global multi-space collaborative computation with self-supervision. Zhang et al. [40] introduced CFPLM, which improves protein–RNA interaction prediction by combining protein language-model embeddings with GCN.

Jha et al. [41] introduced a method that integrates GNNs with language models to predict PPIs. Their approach involves constructing protein graphs from PDB structures and processing LM-derived feature vectors through GNNs for PPI classification. Koca et al. [42] proposed a GraphSAGE-based framework that leverages hybrid embeddings derived from amino acid sequences to predict human-virus protein interactions. Xiao et al. [43] presented HO-VGAE, a Higher-Order GCN Variational Auto-Encoder that combines GCNs with PageRank to learn joint nodes. Li et al. [44] enhanced deep GCNs with residual and dense connections, incorporating dilated convolutions for improved PPI detection. Voytetskiy et al. [45] conducted a comparative study of GNN models such as GCN and GAT for PPI prediction. Zhu et al. [46] applied a semi-supervised GCN model for PPI and protein complex detection. Table 2 evaluates the papers.

### 4.2. Deep Neural Networks (DNNs) Technique

#### 4.2.1. Components and Rationale of DNNs for PPI Prediction

**Figure 3:**
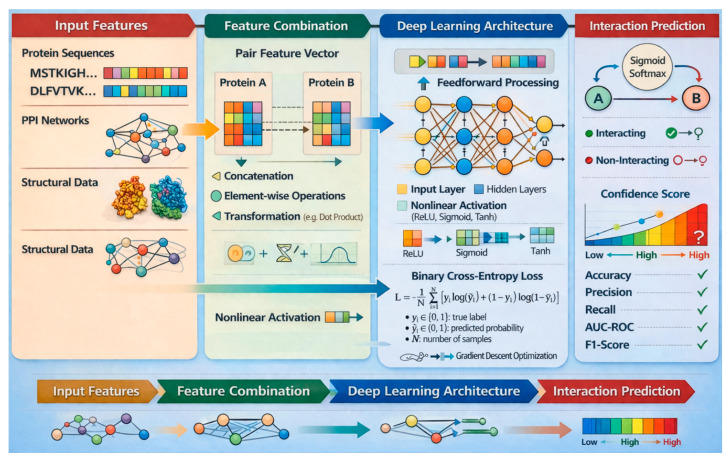
DNNs for PPI Prediction. Multiple input modalities are first extracted and transformed into numerical feature vectors. For a protein pair (Protein A and Protein B), these representations are combined into a joint pairwise feature vector using operations such as concatenation, element-wise multiplication, subtraction, or similarity transformations. The combined feature vector is then fed into a deep feedforward neural network consisting of multiple fully connected hidden layers with nonlinear activation functions enabling hierarchical feature abstraction and modeling of complex nonlinear interaction patterns. During training, the network parameters are optimized using backpropagation with a binary cross-entropy loss function and gradient-based optimization. The final output layer produces a binary interaction prediction (interacting vs. non-interacting) along with a confidence score.

DNNs are composed of an input layer, multiple hidden layers, and an output layer. Each hidden layer consists of neurons that perform nonlinear transformations on the input data through weighted connections and activation functions such as ReLU or sigmoid. For PPI prediction, protein features—such as amino acid compositions, sequence embeddings, or structural descriptors—are fed into the network to learn complex representations. The final output layer typically applies a sigmoid or softmax function to classify whether a protein pair interacts or not. DNNs are particularly effective for PPI prediction due to their ability to model non-linear and high-dimensional relationships inherent in biological data. They automatically learn hierarchical feature representations from raw or engineered inputs, eliminating the need for manual feature design. This makes them well-suited for capturing intricate dependencies between proteins that traditional models might overlook. DNNs can scale large datasets and experimental conditions, improving robustness. Figure 3 depicts the DNN structure.

#### 4.2.2. Mathematical Formulation of DNNs

Let $W^{\left(l\right)}$ and $b^{\left(l\right)}$ be the weight matrix and bias vector for layer l, respectively. For input vector $x_{i,j}$, the output at layer l is as shown in Equation (3):

(3) $$h^{\left(l\right)}=\sigma \left(W^{\left(l\right)}h^{\left(l-1\right)}+b^{\left(l\right)}\right)$$

- $h^{\left(l-1\right)}$ is the output of the previous layer $\left(l-1\right)$ or the input vector $x_{i,j}$ for the first hidden layer.
- $\sigma \left(\cdot \right)$ is the activation function, commonly the ReLU (Rectified Linear Unit), defined as: $\sigma \left(x\right)=\max\left(0,x\right)$.

The final layer is a fully connected layer that maps the last hidden layer’s output to a scalar score or probability representing the likelihood of interaction between the two proteins. This is done through a softmax function for classification as shown in Equation (4):

(4) $$y_{i,j}=\mathrm{softmax}\left(W^{\left(L\right)}h^{\left(l-1\right)}+b^{\left(l\right)}\right)$$

where L is the total number of layers, and $y_{i,j}$ is the predicted probability that the proteins $P_{i}$ and $P_{j}$ interact.

#### 4.2.3. Featuring and Evaluating Research Papers That Have Utilized DNNs for PPI Detection

Dey et al. [47] proposed a supervised-learning framework to predict Ebola virus protein targets and PPI by training multiple classifiers (including deep multi-layer perceptron and classical ML models) on curated interaction evidence. Gainza et al. [48] synthesized strengths, limitations, and practical considerations for PPI prediction pipelines rather than introducing a single new supervised model or benchmark result. Samarasinghe et al. [49] introduced Auto-Associative Neural Networks as a data-driven way to model proteins and PPI networks, using neurons to represent proteins and interaction mechanisms aligned with network topology. Zhang et al. [50] proposed MCLPPII, a multimodal contrastive-learning framework for predicting PPI inhibitors (PPIIs), combining SMILES sequence modeling with multiple molecular fingerprint modalities and a three-stage training strategy. Shao et al. [51] proposed ProFun-SOM for protein function prediction, using multiple sequence alignments and a self-organizing map (SOM) to learn compact, informative protein representations and improve downstream classification. Qiu et al. [52] proposed DrugProtKGE, a weakly supervised approach that fine-tunes triple embeddings by leveraging similarity signals derived from pre-trained knowledge graph embeddings and a Siamese-like neural architecture.

Kumar et al. [53] proposed a deep learning approach for predicting PPIs using an Artificial Neural Network (ANN) implemented with TensorFlow, Keras, and Scikit-learn, optimizing performance across various algorithms, including k-Nearest Neighbor, Logistic Regression, Decision Tree, Random Forest, and Support Vector Machine. Li and Yu [54] introduced a hybrid deep neural network architecture that integrates convolutional and recurrent layers for sequence transformation and contextual feature extraction. Li et al. [55] developed a self-attention-based neural model for PPI prediction, utilizing AAC, CT, and AC descriptors to encode sequence information and dynamically reweight sequence features. Wang et al. [56] improved PPI prediction accuracy by refining the parameters of deep neural networks. Tran et al. [57] combined deep learning with feature fusion techniques by integrating handcrafted features and sequence embeddings for enhanced PPI prediction. Vyas et al. [58] employed a Genetic Programming-based Symbolic Regression approach alongside an ANN to identify disease-associated PPIs using cancer-specific datasets. Bakar et al. [59] utilized ANN to predict yeast PPIs by incorporating features such as secondary structures, co-localization, and protein function. Goodacre et al. [60] applied ANN and Random Forest models to detect disruptive non-synonymous SNPs in HIV-1 and human protein interactions. Table 3 evaluates the above papers.

### 4.3. Convolutional Neural Networks (CNNs) Technique

#### 4.3.1. Components and Rationale of CNNs for PPI Prediction

CNNs are composed of several core components: convolutional layers, pooling layers, activation functions, and fully connected layers. The convolutional layers apply filters to capture local patterns and features within protein sequences or structural data. Pooling layers, such as max-pooling, reduce the spatial dimensions of the feature maps, making the model more computationally efficient and robust. Activation functions like ReLU introduce non-linearity, and fully connected layers integrate the extracted features to perform the final classification or interaction prediction.

**Figure 4:**
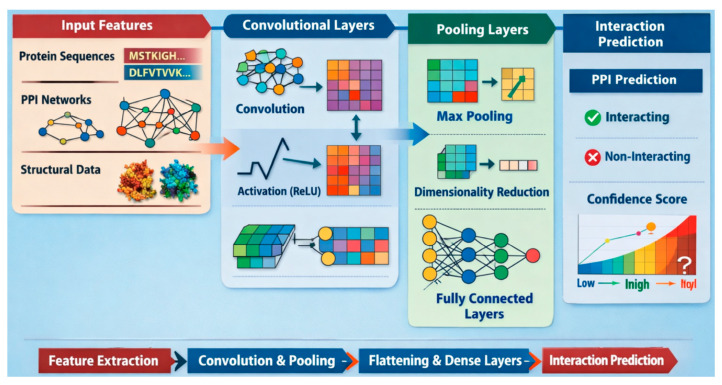
CNNs for PPI Prediction. Protein pairs are first represented through multiple input modalities, including primary amino acid sequences (e.g., one-hot encoding, physicochemical property vectors, or embedding representations), interaction network topology features, and optional structural descriptors. These representations are organized into fixed-length matrices or multi-channel tensors and fed into convolutional layers, where learnable kernels extract local sequence motifs, spatial patterns, and interaction-relevant features. The convolution operations are followed by nonlinear activation functions to introduce model expressiveness and mitigate vanishing gradients. Pooling layers (e.g., max pooling) perform dimensionality reduction while preserving the most discriminative features, enhancing translation invariance and reducing overfitting. The resulting feature maps are flattened and passed to fully connected layers that integrate high-level representations across both proteins. Finally, a sigmoid or softmax output layer produces the PPI prediction along with a confidence score, which was trained using a binary cross-entropy loss optimized via backpropagation.

CNNs are well-suited for PPI prediction due to their ability to automatically learn and extract meaningful patterns from raw biological data. Protein sequences and structures often contain complex local motifs and global dependencies, which CNNs can effectively model through hierarchical feature extraction. Unlike traditional models, CNNs do not require extensive manual feature engineering, enabling them to generalize well across diverse protein datasets. Their effectiveness in capturing spatial and sequential dependencies makes them a powerful approach for accurate and scalable PPI prediction. Figure 4 depicts the processing of a CNN.

#### 4.3.2. Mathematical Formulation of CNNs

The convolutional layer applies a filter (kernel) $W_{c}\in {\mathbb{R}}^{f\times d}$ across the input matrix, where *f* is the filter size (or receptive field) and *d* is the depth of input channels. Equation (5) defines the convolution operation:

(5) $$h_{i,j}^{\left(1\right)}=\sigma \left(\sum_{a=1}^{f}\sum_{b=1}^{d}W_{c}\left[a,b\right]\cdot X_{i+a-1,j+b-1}+b_{c}\right)$$

where $h_{i,j}^{\left(1\right)}$ is the activation for the first convolutional layer, σ is the activation function (commonly ReLU), and $b_{c}$ is the bias term. This results in a feature map $H^{\left(1\right)}\in {\mathbb{R}}^{\left(m-f+1\right)\times \left(n-d+1\right)}$.

After convolution, a pooling layer (e.g., max pooling) reduces the dimensionality of the feature map to focus on the important features. Pooling is performed over a window size P×P, defined as Equation (6):

(6) $$h_{i,j}^{\left(2\right)}=\underset{a,b\in \left[0,p-1\right]}{\lim}h_{i+a,j+b}^{\left(1\right)}$$

The output of the pooling layer is a down-sampled feature map, as shown in Equation (7):

(7) $$H^{\left(2\right)}\in {\mathbb{R}}^{\frac{m}{p}\times \frac{n}{p}}$$

After several convolution and pooling layers, the feature map is flattened into a vector $h^{\left(f\right)}\in {\mathbb{R}}^{l}$, where l is the length of the flattened vector. This vector is passed through a fully connected layer with weight matrix $W_{fc}\in {\mathbb{R}}^{o\times l}$ and bias term $b_{fc}\in {\mathbb{R}}^{o}$. The output of the fully connected layer is passed through a softmax function to get the probability distribution for classifying the interaction as positive (interacting) or negative (non-interacting) as shown in Equation (8):

(8) $$P\left(y_{i}=c|X_{i}\right)=\frac{e^{zc}}{\sum_{j=1}^{o}e^{zj}}$$

where $P\left(y_{i}=c|X_{i}\right)$ is the probability that the input interaction $X_{i}$ belongs to class *c*.

#### 4.3.3. Featuring and Evaluating Research Papers That Have Utilized CNNs for PPI Detection

Han et al. [61] introduced PPI-BAN, which is an end-to-end framework that predicts whether two proteins interact by integrating sequence features extracted with Conv1D and 3D-structure features learned from AlphaFold2-derived graphs. Tang et al. [62] introduced CoMPPI, which addresses multi-label PPI type prediction by co-training two complementary views of sequence information: ordered (contextual) features processed with a 1D CNN (textCNN). Bi et al. [63] introduced SSPPI, which is a multimodal deep learning framework that builds richer protein representations by combining sequence and structure encoders (Convformer and Graphormer). Paul et al. [64] proposed generative deep learning models (e.g., variational autoencoders) to generate protein sequences from large unlabeled data.

Gao et al. [65] proposed EResCNN, an ensemble residual convolutional neural network designed for reliable PPI prediction by incorporating diverse features such as physicochemical properties and evolutionary information. Hu et al. [66] introduced DeepTrio, a deep learning framework leveraging multiscale CNNs to extract contextual insights from protein sequences. Xie et al. [67] built a CNN-based model to predict PPI sites. Cai and Zhu [68] employed particle swarm optimization (PSO) to construct a deep CNN for identifying essential proteins. Zhang et al. [69] designed a residual CNN aimed at PPI extraction. Yuan et al. [70] presented a deep transfer learning-based model for PPI prediction. Dutta et al. [71] developed an ensemble clustering approach, combining a multi-layer perceptron and CNN for both PPI and gene. The above papers are evaluated in Table 4.

### 4.4. Extreme Learning Machine (ELM) Technique

#### 4.4.1. Components and Rationale of ELM for PPI Prediction

ELM is a feedforward neural network model primarily composed of an input layer, a single hidden layer with randomly assigned weights, and an output layer. Unlike traditional neural networks, ELM does not require iterative tuning of weights; instead, it analytically determines output weights using least squares optimization. The hidden layer’s parameters remain fixed, which significantly accelerates the training process. This architecture allows ELM to efficiently handle high-dimensional input data, such as protein sequences used in PPI prediction.

**Figure 5:**
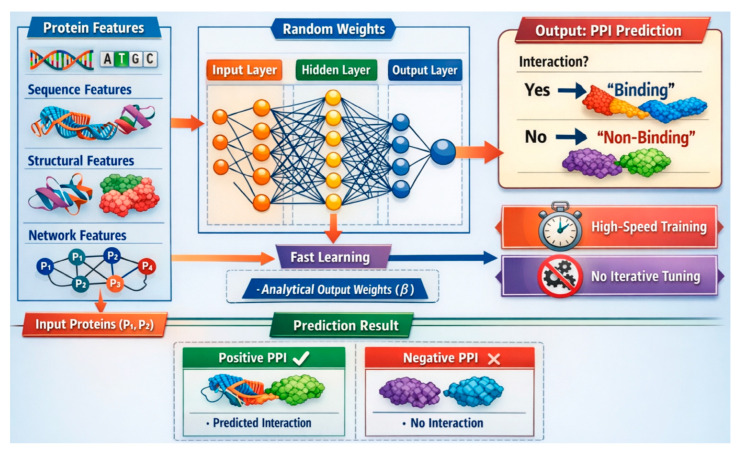
**Architecture and processing pipeline of ELM for PPI prediction.** First, heterogeneous protein representations—including sequence-derived features (e.g., k-mer composition, physicochemical descriptors), structural features (e.g., secondary structure, solvent accessibility), and network/topological features—are extracted for a protein pair $\left(P_{1},P_{2}\right)$ and concatenated into a fixed-length input vector. These features are fed into a single-hidden-layer feedforward neural network where input weights and hidden biases are randomly initialized and remain fixed. The hidden layer performs a nonlinear transformation using activation functions (e.g., sigmoid, ReLU), generating the hidden layer output matrix H. Unlike conventional neural networks, ELM analytically computes the output weight matrix β using the Moore–Penrose pseudoinverse ($\beta =H^{†}T$), enabling fast training without iterative backpropagation. The final output layer produces binary classification results that indicate positive (binding) or negative (non-binding) interactions.

ELM is particularly advantageous for PPI prediction due to its fast learning capability and strong generalization performance. Biological datasets, especially in PPI studies, are often large and complex, making training time a critical factor. ELM’s ability to learn from data with minimal computational cost makes it suitable for real-time or large-scale PPI analysis. Its analytical training approach avoids issues like local minima, offering a stable and scalable solution for predicting protein interactions. Figure 5 depicts the ELM structure.

#### 4.4.2. Mathematical Formulation of ELM

Given a dataset with *N* protein–protein interaction samples as shown in Equation (9):

(9) $$\left\{\left(x_{i},t_{i}\right)|x_{i}\in {\mathbb{R}}^{n},t_{i}\in {\mathbb{R}}^{m},i=i,1,2,\ldots ,N\right\}$$

where *x_i_* is the feature vector representing a protein pair, and *t_i_* is the corresponding label (e.g., interaction or no interaction). The output of the hidden layer is computed as shown in Equation (10):

(10) $$H=q\left(W_{x}+b\right)$$

- $W\in {\mathbb{R}}^{L\times n}$: Random weights between the input and hidden layer.
- $b\in {\mathbb{R}}^{L}$: Random biases for the hidden layer nodes.
- *g*(⋅): Activation function (e.g., sigmoid, ReLU, or radial basis function).
- $H\in {\mathbb{R}}^{N\times L}$: Hidden layer output matrix.

The relationship between the hidden layer and the output is as shown in Equation (11):

*H* *β* = *T* (11)

- $\beta \in {\mathbb{R}}^{L\times m}$: Output weights between the hidden layer and the output layer.
- $T\in {\mathbb{R}}^{N\times m}$: Target matrix derived from *t_i_*.

The ELM determines *β* by minimizing the following optimization shown in Equation (12):

(12) $$\underset{\beta }{\min}={\left\|H\beta -T\right\|}_{2}$$

The solution is given in closed form as shown in Equation (13):

(13) $$\beta =H^{†}\;T$$

- $H^{†}$ is the Moore–Penrose generalized inverse of *H*, computed as:
- $H^{†}={\left(H^{T}\;H\right)}^{-1}H^{T}\;\mathrm{if}\;H^{T}\;H$ is nonsingular.
- $H^{†}=H^{T}{\left(H\;H^{T}\right)}^{-1}\;\mathrm{if}\;H\;H^{T}$ is nonsingular.

#### 4.4.3. Featuring and Evaluating Research Papers That Have Utilized ELM for PPI Detection

Deddy et al. [72] introduced a PPI prediction algorithm that uses the ELM algorithm, achieving effective results. You et al. [73] proposed an ELM-based PPI prediction technique focused on local protein sequence descriptors, extracting more PPI details from sequences. Sikandar et al. [74] explored computational techniques for disease-gene association by understanding PPI within the human genome, using topological features. You et al. [75] presented a Low Approximation Kernel LELM framework for human PPI detection from primary sequences. Table 1 evaluates the above research papers.


**Comparative Insights Across Neural Network-Based Learning PPI Detection Families: ELM, CNN, GNN, vs. generic DNN/Transformers**
  ***ELM* vs. *CNN:*** For sequence-only PPI pipelines, ELM variants (e.g., PCA-EELM, WELM-SURF) train in seconds and handle class imbalance cheaply, but they rely on hand-crafted k-mer/physicochemical features and plateau on cross-species transfer; CNNs learn discriminative local motifs from raw sequences (e.g., residual/ensemble CNNs) and typically surpass ELMs on accuracy, albeit with higher compute and careful regularization.  ***CNN vs. GNN:*** CNNs excel when only primary sequence is available, and labeling is noisy, but they miss 3D geometry and long-range contacts; GNNs built on residue/atom graphs integrate sequence and structure, capturing spatial dependencies and boosting generalization on structure-rich benchmarks.  ***GNN* vs. *generic DNN/Transformers:*** Modern DNN stacks using protein language models (PLMs) provide powerful per-protein embeddings for pairwise classification, yet they can conflate evolutionary signal with data leakage and struggle without explicit interface cues; GNNs complement PLMs by message passing over contact graphs or PPI networks, improving interface-aware predictions and multi-category interaction types. PLMs generate context-aware protein embeddings, which capture evolutionary, structural, and functional information without manual feature engineering. In PLM-interact frameworks: (1) each protein is encoded independently using a PLM; (2) the resulting embeddings are combined (e.g., concatenation, similarity scoring); (3) a downstream classifier predicts interaction probability. PLM-based models significantly improve performance due to rich representations. However, they may suffer from data leakage risks and lack of explicit structural reasoning, which motivates hybrid approaches.  ***ELM* vs. *DNN (deployment):*** If you need rapid screening over millions of candidate pairs or edge devices in labs, ELMs offer excellent latency/throughput and transparent calibration but require curated features and careful negative sampling; DNNs/PLMs are state-of-the-art in accuracy and cross-domain transfer, yet demand GPUs, larger training sets, and vigilance against train–test homology bias.   ***End-to-end takeaway (real-world PPI detection):*** For sequence-only, scarce-compute settings choose CNNs (or ELMs for ultrafast triage); for structure-available or interface-critical tasks choose GNNs; and for maximal accuracy under ample compute, use PLM-based DNN—hybrid PLM→GNN models that join learned embeddings with graph.

## 5. Margin-Based Discriminative Category

### 5.1. Support Vector Machine (SVM) Technique

#### 5.1.1. Components and Rationale of SVM for PPI Prediction

SVM is a supervised learning algorithm that constructs a hyperplane to separate data points into different classes with maximum margin. In PPI prediction, input features—such as amino acid composition, evolutionary profiles, or structural descriptors—are mapped into a high-dimensional space using kernel functions (e.g., RBF) or polynomial kernels. The algorithm then identifies the optimal hyperplane that maximally distinguishes interacting from non-interacting protein pairs. Support vectors, which are the data points closest to the decision boundary, play a crucial role in defining this hyperplane.

**Figure 6:**
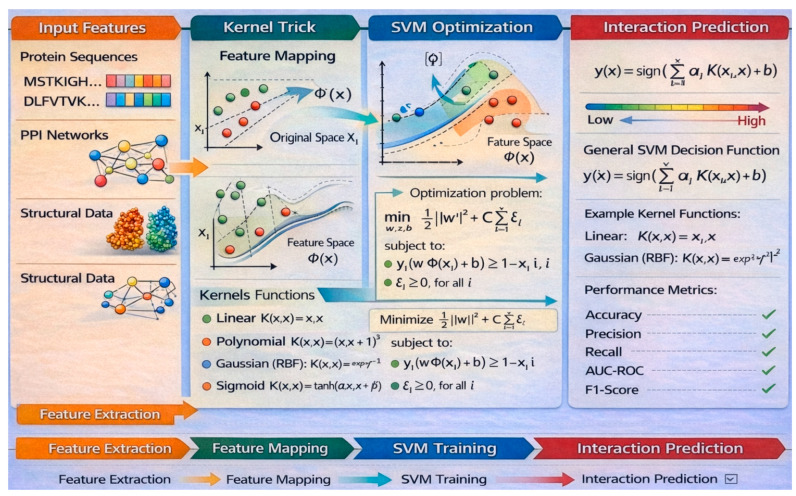
**SVM for PPI Prediction.** Protein pairs are encoded using sequence, structural, and network-derived features and labeled as interacting (+1) or non-interacting (−1). To address nonlinear separability, kernel functions (e.g., linear, polynomial, RBF) map features into a higher-dimensional space. The model learns an optimal separating hyperplane by solving a margin-maximization problem with slack variables, and predictions are made using the decision function $\hat{y}(x)=\mathrm{sign}\left(\sum_{i}\alpha_{i}y_{i}K(x_{i},x)+b\right).$ The output includes the interaction label and a confidence score (distance from the hyperplane).

SVM is particularly effective for PPI prediction due to its ability to handle high-dimensional, non-linear biological data through kernel transformations. It offers robust generalization even when the data is imbalanced or noisy, a common scenario in biological datasets. The model’s flexibility in choosing kernels allows it to capture complex patterns in protein interactions. SVMs often outperform traditional models in terms of accuracy and are well-suited for both binary and multi-class PPI classification tasks. Figure 6 illustrates the procedure of the SVM.

#### 5.1.2. Mathematical Formulation of SVM

The following is the mathematical formulation of the SVM. Given a labeled dataset ${\left\{\left(x_{i},y_{i}\right)\right\}}_{i=1}^{N}$:

- $x_{i}\in {\mathbb{R}}^{d}$: feature vector representing a protein pair.
- $y_{i}\in \left\{-1,+1\right\}$: label indicating whether the pair interacts (+1) or does not interact (−1).

The SVM optimization problem is defined as shown in Equation (14):

(14) $$\underset{w,b,\xi }{\min}\frac{1}{2}{\left\|w\right\|}^{2}+C\sum_{i=1}^{N}\xi_{i}$$

Subject to Equation (15):

(15) $$y_{i}\left(w^{Τ}x_{i}+b\right)\geq 1-\xi_{i},\xi_{i}\geq 0,\forall i\in \left\{1,\ldots ,N\right\}$$

- *w*: normal vector to the hyperplane.
- *b*: bias term.
- $\xi_{i}$: slack variable for each data point, allowing for soft margin classification.
- *C* > 0: regularization parameter controlling the trade-off between maximizing the margin and minimizing classification error.

To handle non-linear relationships in PPI, SVM uses kernel functions *K*(x_i_, x_j_) to map data into a higher-dimensional feature space as shown in Equation (16):

(16) $$K\left(x_{i},x_{j}\right)=\phi {\left(x_{i}\right)}^{Τ}\phi \left(x_{j}\right)$$

Common kernels include Linear Kernel as shown in Equation (17):

(17) $$K\left(x_{i},x_{j}\right)=x_{i}^{Τ}x_{j}$$

The decision function for a new data point *x* is as shown in Equation (18):

(18) $$f\left(x\right)=\mathrm{sign}\left(\sum_{i=1}^{N}\alpha_{i}y_{i}K\left(x_{i},x\right)+b\right)$$

- $\alpha_{i}$: Lagrange multipliers from the dual optimization problem.
- $K\left(x_{i},x\right)$: kernel function applied to the new data point and support vectors.

#### 5.1.3. Featuring and Evaluating Research Papers That Have Utilized SVM for PPI Detection

Wang et al. [76] propose ELF-DPC, an ensemble learning framework for detecting protein complexes in PPI networks by integrating biological information (GO, gene expression, localization) with topological features and a voting regression model combined with structural modularity. Choppara et al. [77] introduced a Quantum Support Vector Machine model for predicting compound–protein interactions in drug discovery, leveraging quantum feature mapping and quantum kernels to enhance nonlinear pattern learning.

Chen et al. [78] employed SVMs and Conditional Random Fields (CRFs) to extract PPI information from text, demonstrating the potential of hybrid approaches for PPI extraction. Lin and Zhang [79] implemented an ensemble SVM method to identify PPI hot spots, effectively enhancing prediction accuracy by integrating multiple SVM models. Chen et al. [80] leveraged Graphics Processing Units (GPUs) to optimize SVM hyperparameter estimation, significantly accelerating computations for PPI datasets. Chatrabgoun et al. [81] utilized SVMs for diverse PPI prediction tasks, showcasing their adaptability across various biological contexts. Sunggawa et al. [82] combined SVM with Mutual Maximum Information (MMI) to predict HIV-1 and human PPIs, demonstrating the effectiveness of integrating SVM with advanced statistical measures for domain-specific PPI analysis. Table 5 evaluated the above papers.

### 5.2. Least Squares SVM (LS-SVM) Technique

#### 5.2.1. Components and Rationale of LS-SVM for PPI Prediction

**Figure 7:**
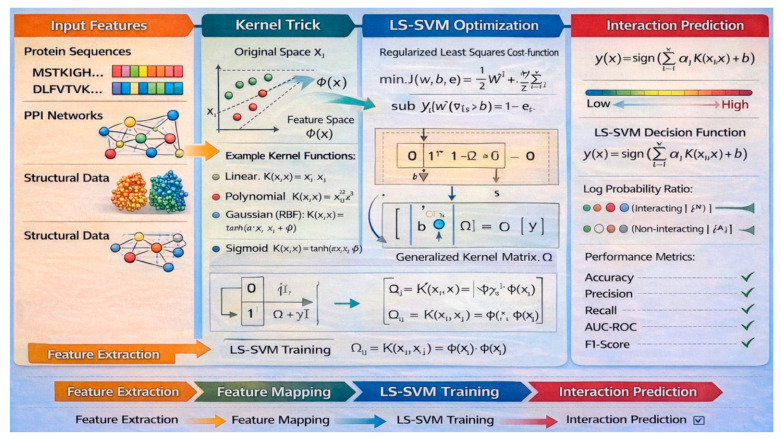
**LS-SVM for PPI Prediction.** The figure depicts the LS-SVM framework for PPI prediction. Protein pairs are encoded using sequence, structural, and network-derived features and mapped into a high-dimensional space via kernel functions (e.g., linear, polynomial, RBF). Unlike standard SVM, LS-SVM solves a regularized least-squares problem with equality constraints, leading to a system of linear equations based on the kernel matrix. The decision function $\hat{y}(x)=\mathrm{sign}\left(\sum_{i}\alpha_{i}K(x_{i},x)+b\right)$ classifies protein pairs as interacting or non-interacting, with confidence derived from the decision value.

LS-SVM is a modified version of traditional SVM that simplifies the optimization process by converting the quadratic programming problem into a set of linear equations. It consists of components such as the input feature space, a kernel function to map inputs into higher-dimensional space, and regularization parameters to control model complexity. Unlike standard SVM, LS-SVM uses a least-squares cost function and equality constraints, which makes the training process computationally efficient. This enables LS-SVM to model complex, non-linear relationships in data using a simplified mathematical structure (Figure 7).

LS-SVM is well-suited for PPI prediction due to its ability to handle noisy and high-dimensional biological data with reduced computational burden. The technique offers a balance between prediction accuracy and computational efficiency, which is especially important when working with large-scale or sparse PPI datasets. Its closed-form solution facilitates faster training without sacrificing the ability to model non-linear interactions among protein features. LS-SVM demonstrates competitive performance in PPI studies.

#### 5.2.2. Mathematical Formulation of LS-SVM

1. *Objective Function*

LS-SVM solves the following optimization problem shown in Equation (19):

(19) $$\underset{w,b,e}{\min}\frac{1}{2}{\left\|w\right\|}^{2}+\frac{\gamma }{2}\sum_{i=1}^{N}e_{i}^{2}$$

subject to:

$$y_{i}=w^{T}\phi (x_{i})+b+e_{i},\;i=1,\ldots ,N$$

where (1) *x_i_*: protein feature vector, (2) *y_i_*: interaction label (+1 or −1), (3) *ϕ*(*x_i_*): feature map to higher-dimensional space, (4) *e_i_*: error term, and (5) *γ*: regularization parameter.

2. *Dual Formulation:* The dual problem leads to a linear system:

$$\left[\begin{array}{cc} 0 & 1^{T}\\ 1 & \Omega +\gamma^{-1I} \end{array}\right]\left[\begin{array}{l} b\\ \alpha \end{array}\right]=\left[\begin{array}{l} 0\\ y \end{array}\right]$$

- Ω_ij_ = *K*(*x_i_*, *x_j_*): kernel matrix.
- *K*(⋅,⋅): kernel function (e.g., RBF, polynomial).

3. *Decision Function is as shown in Equation (20):*

(20) $$f(x)\sum_{i=1}^{N}\alpha_{i}K(x_{i},x)+b$$

This computes the likelihood of a protein pair interacting based on kernel similarity and learned weights.

#### 5.2.3. Featuring and Evaluating Research Papers That Have Utilized LS-SVM for PPI Detection

Zhang et al. [83] propose a method for PPI hot spots, which uses LS-SVM within a Bayesian inference framework. By incorporating a three-level Bayesian approach, the model efficiently optimizes LS-SVM parameters and eliminates the need for cross-validation. Ji-Yong et al. [84] introduce a computational method, RVMAB, for PPI prediction by combining Relevance Vector Machine classification with Average Blocks feature extraction (Table 6).




**Comparative Insights Across Margin-Based Families: SVM vs. Least Squares SVM**

***Optimization and model footprint:*** For PPI detection with heterogeneous descriptors (e.g., PSSM, contact potentials, structure cues), classic SVM’s hinge-loss maximizes a geometric margin and yields *sparse* solutions (few support vectors), so inference on proteome-scale screens is lean; LS-SVM’s least-squares reformulation solves linear systems quickly but produces *denser* decision functions, trading faster retraining for heavier prediction cost. This makes SVM preferable when you must score billions of pairs or deploy on constrained hardware, while LS-SVM is advantageous when gold standards or features change frequently and you must refit often. ***Noise, imbalance and calibration:*** Under real assay noise and severe class imbalance (true interactions are rare), SVM’s margin objective is slightly more outlier-tolerant, whereas LS-SVM’s quadratic loss amplifies the influence of mislabeled positives; with reweighting/SMOTE, both can recover minority recall, but SVM typically preserves precision better at the same recall. For probabilistic scoring and automated hyperparameter selection, LS-SVM integrates naturally with evidence/Bayesian formulations, while standard SVM usually needs post-hoc calibration (Platt/isotonic) and broader C–γ searches; so LS-SVM gives smoother probability estimates out-of-the-box, whereas SVM gives sharper decision boundaries that generalize more stably under label noise. ***Feature–kernel interplay and deployment roles:*** With string, spectrum, or RBF kernels over sequence/structure features, both families reach similar peak accuracy on moderate-sized PPI sets; the practical gap emerges in *how* they get there—SVM’s sparsity curbs latency at scale, while LS-SVM’s linear-algebra training accelerates iteration on new organisms or feature sets. In production pipelines, a high-throughput pattern is to use LS-SVM for rapid model refreshes and calibrated candidate scoring, then freeze a classic SVM for the final, sparse reranker that meets throughput/SLA targets; invert that choice only if retraining frequency dominates runtime cost. Overall, choose LS-SVM when you value swift, well-calibrated retraining and SVM when you need durable margins, fewer support vectors, and predictable speed at deployment.


## 6. Probabilistic Learning Category

### 6.1. Naïve Bayes Technique

#### 6.1.1. Components and Rationale of Naïve Bayes for PPI Prediction

The Naïve Bayes technique is a probabilistic classifier based on Bayes’ Theorem, assuming independence among features. In the context of PPI prediction, input features such as amino acid composition, physicochemical properties, or sequence patterns are used to compute class probabilities. The model estimates the likelihood of a protein pair interacting by combining the prior probabilities of interaction with the conditional probabilities of observed features. Despite its simplicity, Naïve Bayes performs well when feature independence approximations are reasonably valid across PPI datasets.

**Figure 8:**
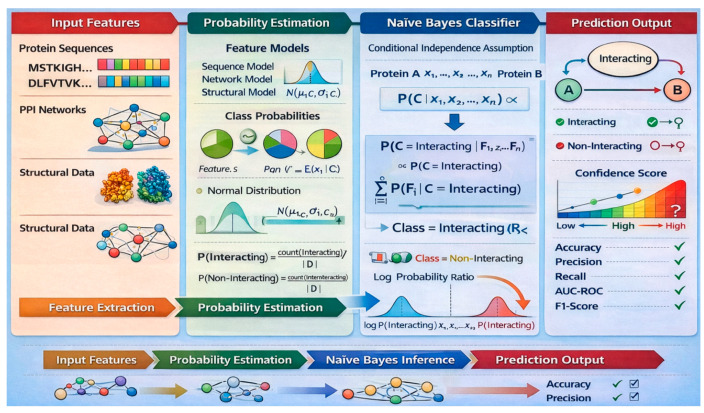
**Naïve Bayes for PPI Prediction.** Protein pairs are first represented through extracted features derived from amino acid sequences (e.g., k-mer frequencies, physicochemical descriptors), PPI network topology metrics (e.g., degree, clustering coefficient), and structural attributes. During probability estimation, class priors P(C) (Interacting vs. Non-Interacting) are computed from training data, while feature likelihoods $P(F_{i}|C)$ are modeled using appropriate distributions (e.g., Gaussian for continuous features or multinomial for discrete features). Under the conditional independence assumption, the posterior probability is computed as $P(C|F_{1},\ldots ,F_{n})\propto P(C)\prod_{i=1}^{n}P(F_{i}|C),$ which is typically evaluated in log-space to avoid numerical underflow. The classifier assigns the class with the maximum posterior probability. The final output indicates whether the protein pair is predicted to be interacting or non-interacting, along with a confidence score derived from posterior probabilities.

Naïve Bayes is well-suited for PPI prediction due to its efficiency in handling high-dimensional biological data and minimal training requirements. It offers fast computation and robust performance even with relatively small datasets, making it ideal for large-scale or preliminary screening tasks. The probabilistic output allows easy interpretation of confidence in predicted interactions, which is valuable for downstream biological validation. Naïve Bayes is easily integrated with other models or ensemble frameworks, which enhances its applicability in diverse PPI prediction scenarios. Figure 8 shows the procedure of Naïve Bayes.

#### 6.1.2. Mathematical Formulation of Naïve Bayes

The fundamental principle of Naïve Bayes is Bayes’ theorem, as shown in Equation (21):

(21) $$P\left(y|X\right)=\frac{P\left(X|y\right)\cdot P\left(y\right)}{P\left(X\right)}$$

where (1) *P*(*y* | *X*): Posterior probability of label *y* (interaction or no interaction) given the feature set *X*, (2) *P*(X | y): Likelihood of observing the feature set *X* given *y*, (3) *P*(*y*): Prior probability of *y*, (4) *P*(*X*): Evidence or marginal probability of the feature set *X*, and (5) *P*(*X*) can be ignored for classification purposes since it is constant for all *y*.

Naïve Bayes assumes that the features *x*_1_, *x*_2_, …, *x_n_* in the feature set *X* are conditionally independent given the class *y*. Therefore (Equation (22)):

(22) $$P\left(X|y\right)=\prod_{i=1}^{n}P\left(x_{i}|y\right)$$

*x_i_* represents individual features derived from properties of the proteins, such as sequence information. The probability that a pair of proteins interacts (*y* = 1) is computed as shown in Equation (23):

(23) $$P\left(y=1|X\right)=\frac{P\left(y=1\right)\cdot \prod_{i=1}^{n}P\left(x_{i}|y=1\right)}{\sum_{y^{\prime }}P\left(y=y^{\prime }\right)\cdot \prod_{i=1}^{n}P\left(x_{i}|y=y^{\prime }\right)}$$

To avoid numerical underflow when computing probabilities for a large number of features, the log form is often used as shown in Equation (24):

(24) $$\log P\left(y|X\right)=\log P\left(y\right)+\sum_{i=1}^{n}\log P\left(x_{i}|y\right)$$

- This linear combination of log probabilities simplifies computation and is widely implemented in practice.

The final classification is made by selecting the class with the highest posterior probability (Equation (25)):

(25) $$y^{*}=\arg\underset{y\in \left\{0,1\right\}}{\max}P\left(y\right)\cdot \prod_{i=1}^{n}P\left(x_{i}|y\right)$$

#### 6.1.3. Featuring and Evaluating Research Papers That Have Utilized Naïve Bayes for PPI Detection

Kong et al. [85] introduced AutoTarget, a novel pipeline that employs node representation learning and a Naïve Bayes classifier to identify druggable targets within PPI networks. Integrating data from the STRING and DisGeNET databases, it embedded proteins into a 128-dimensional space, uncovering 3979 targets mapped to 23,363 diseases. Xu et al. [86] tackled unreliable interactions in protein complexes using Fuzzy Naïve Bayes. They focused on classifying protein complexes from subgraphs, considering PPI’s fuzzy nature. Metipatil et al. [87] used machine learning, including Naïve Bayes and SVM, to classify cancer gene data based on binary PPI and gene expressions. Hu et al. [88] proposed an ML-based method to predict PPI hotspots, using variables like amino acid composition. They utilized Gaussian Naïve Bayes for prediction. Table 7 evaluates the above papers.

### 6.2. Probabilistic Decision Tree Technique

#### 6.2.1. Components and Rationale of Probabilistic Decision Tree for PPI Prediction

The Probabilistic Decision Tree technique consists of internal decision nodes, probabilistic branching, and leaf nodes with class distribution estimates. Each internal node evaluates a specific protein feature—such as sequence similarity, structural properties, or physicochemical attributes—using probabilistic thresholds rather than hard splits. At each node, instead of making a binary decision, the model assigns probabilities to branches based on the likelihood of feature values. The leaf nodes provide probability distributions over class labels, allowing the model to output confidence scores.

**Figure 9:**
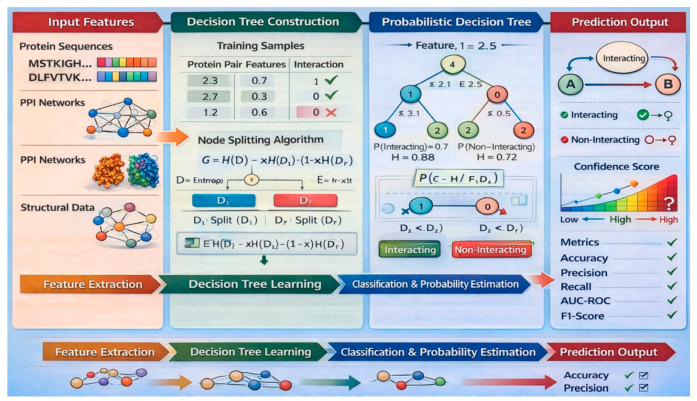
Probabilistic Decision Tree for PPI Prediction. The figure illustrates a probabilistic decision tree framework for predicting protein–protein interactions. Protein pairs are first represented using extracted features derived from amino acid sequences (e.g., k-mer frequencies, physicochemical properties), PPI network topology metrics (e.g., degree, betweenness, clustering coefficient), and structural descriptors. These features form a pairwise feature vector used as input to the tree construction phase. During training, the decision tree is built using recursive node splitting based on impurity reduction criteria such as entropy $H(D)=-\sum_{c}p(c)\log\;p(c)$, Gini index, or information gain. In the probabilistic decision tree, each leaf node not only assigns a class label (Interacting or Non-Interacting) but also estimates class posterior probabilities P(C∣F) based on the distribution of training samples.

Probabilistic Decision Trees are well-suited for PPI prediction because they handle uncertainty and noise commonly found in biological datasets. Unlike traditional decision trees, they maintain multiple possible paths during prediction, which improves robustness and flexibility. Their probabilistic nature allows for soft classification, making them valuable for ranking potential interactions and supporting biological inference. Furthermore, the interpretable structure of decision trees enables researchers to trace the decision-making process, aiding in the validation of interaction hypotheses. Figure 9 shows this procedure.

#### 6.2.2. Mathematical Formulation of Probabilistic Decision Tree

The following is the mathematical formulation of a probabilistic decision tree. At each internal node *t*, a probabilistic decision is made based on a feature *f_t_* and a probabilistic distribution over the feature values $P\left(f_{t}|X\right)$. Let: (1) $f_{t}\left(X\right)$: The value of feature f for a protein pair *X*; (2) $P_{t}\left(c|X\right)$: The conditional probability of class *c* at node *t*. A probabilistic decision rule routes the sample *X* to child nodes:

(26) $$P_{child\left(t\right)}\left(X\right)=P\left(f_{t}\left(X\right)|X\right)$$

where child(*t*) is the set of possible child nodes. The transition probability from a parent node *t_p_* to a child node *t_c_* is given by Equation (27):

(27) $$P\left(t_{c}|t_{p},X\right)=P\left(f_{t}\left(X\right)=v|X\right)$$

where *v* is the observed value of *f_t_*(*X*).

At each leaf node *L*, the probability of the label *Y* = *c* is given by Equation (28):

(28) $$P_{L}\left(Y=c|X\right)=\frac{\mathrm{Count}\left(c,L\right)}{\mathrm{Count}\left(L\right)}$$

- Count(*c*, *L*): The number of samples of class *c* reaching the leaf node *L*.
- Count(*L*): The total number of samples reaching *L*.

The probability of the label *Y* = *c* for a protein pair *X* is computed by traversing the tree and aggregating probabilities as shown in Equation (29):

(29) $$P\left(Y=c|X\right)=\prod_{T\in \mathrm{path}\left(X\right)}P\left(t|t_{\mathrm{parent},X}\right)$$

where path(*X*) is the sequence of nodes visited by *X*. The final classification is given by Equation (30):

(30) $$\overset{⌢}{Y}=\arg\underset{c\in \left\{0,\;1\right\}}{\max}P\left(Y=c|X\right)$$

#### 6.2.3. Featuring and Evaluating Papers That Utilized Probabilistic Decision Tree for PPI Detection

Cecchini et al. [25] presented a method to predict metabolic disease genes by integrating PPI and miRNA-target interactions, addressing data imbalance. They used topological features and a Probabilistic Decision Tree with boosting. Zaki and Alashwal [89] identified missing links in PPI networks via topological analysis to enhance protein complex identification. They applied a boosted decision-tree classifier for interactome link distinction. Yang et al. [90] used a Probabilistic Decision Tree for PPI scores based on proteins’ CETSA features. Table 8 evaluates the above papers.



**Comparative Insights Across Probabilistic-Based PPI Detection Families: Naïve Bayes vs. Probabilistic Decision Trees**
Naïve Bayes (NB) and Probabilistic Decision Trees (PDTs) are both probabilistic learners used in protein–protein interaction (PPI) detection, but differ in how they handle feature dependencies. NB assumes independence among features, enabling rapid computation and scalability for large proteomic datasets. However, this assumption often fails in biological data where features like residue position and solvent accessibility are correlated, reducing NB’s predictive power. PDTs overcome this by modeling conditional dependencies through hierarchical decision paths, leading to more accurate interaction predictions when feature correlations exist.  NB’s interpretability lies in its clear feature likelihoods, offering quick insights into individual contributions, whereas PDTs produce rule-based explanations that biologists can easily interpret and validate experimentally. Thus, PDTs provide deeper interpretability, while NB favors computational simplicity and speed. Under class imbalance and noisy labeling—common in PPI datasets—PDTs handle skewed data better through resampling or boosting, whereas NB requires careful probability calibration to avoid bias.  NB excels in high-throughput screening due to its linear complexity and minimal training cost, making it suitable for early-stage candidate filtering. PDTs, although more resource-intensive, deliver stronger reliability in final-stage validation where nuanced feature relationships matter. In large-scale deployment pipelines, NB acts effectively as a first-pass filter, while PDTs function as a second-tier model for probabilistic refinement. Overall, NB offers scalability and simplicity, but PDTs achieve greater robustness, interpretability, and accuracy in complex, real-world PPI detection scenarios.


## 7. Instance-Based Learning Category

### 7.1. K-Nearest Neighbor (KNN) Technique

#### 7.1.1. Components and Rationale of KNN for PPI Prediction

The KNN technique is a non-parametric, instance-based learning algorithm that classifies data points based on the majority class among their k-nearest neighbors in the feature space. For PPI prediction, the components of KNN include a distance metric (such as Euclidean or cosine distance), a labeled dataset of protein pairs, a value of *k* defining the number of neighbors to consider, and a voting mechanism to determine the class label. Each protein pair is represented using feature vectors derived from sequence, structural, or functional attributes. The algorithm identifies the *k* closest protein pairs from the training set and assigns the interaction label based on their majority class.

**Figure 10:**
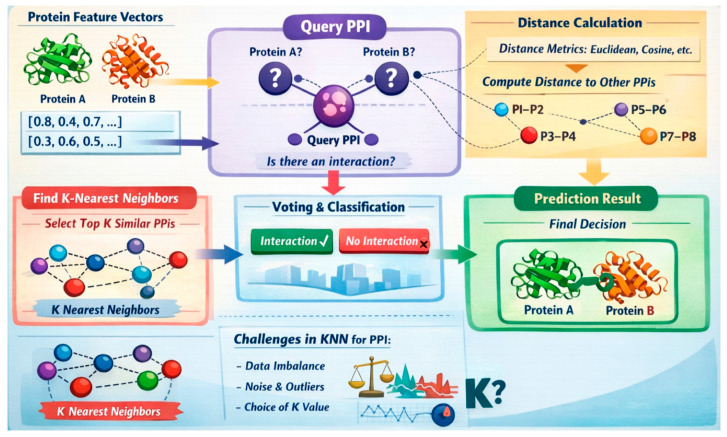
**KNN framework for PPI prediction**. First, proteins are encoded into numerical feature vectors derived from sequence composition, physicochemical properties, structural descriptors, or embedding representations. A query protein pair (Protein A, Protein B) is constructed and represented in the same feature space. Pairwise similarity is computed between the query PPI and labeled training PPIs using distance metrics such as Euclidean or cosine distance. The top-K most similar interaction instances are selected as nearest neighbors. A majority voting scheme is then applied to classify the query pair as interaction or non-interaction. The figure also highlights key technical considerations, including feature representation quality, distance metric selection, class imbalance, noise sensitivity, and the impact of choosing an optimal K value.

The rationale for using KNN in PPI prediction lies in its simplicity, effectiveness, and ability to model complex interaction patterns without explicit assumptions about the data distribution. It leverages the biological principle that similar protein pairs tend to exhibit similar interaction behaviors. By relying on local decision boundaries, KNN can adapt to diverse and high-dimensional feature representations derived from protein data. Its interpretability and minimal training time make it a choice for exploratory PPI analysis. The procedure of KNN is illustrated in Figure 10.

#### 7.1.2. Mathematical Formulation of KNN

The following is the mathematical formulation of KNN:

(1) *Input:* A query protein pair (*q*_1_, *q*_2_), with features $q_{1},q_{2}\in {\mathbb{R}}^{m}$. A dataset of proteins represented as feature vectors, as shown in Equation (31):
  (31) $$D=\left\{\left(x_{1},y_{1}\right),\left(x_{2},y_{2}\right),\ldots ,\left(x_{n},y_{n}\right)\right\}$$
  - $x_{i}\in {\mathbb{R}}^{m}$ is the feature vector of protein *i*,
  - $y_{i}\in \left\{0,1\right\}$ is the label indicating interaction (1)/non-interaction (0)
(2) *Distance Metric: Compute* a distance *d*(*x_i_*, *q*_1_) and d(*x_i_*, *q*_2_) between the query proteins and the proteins in the dataset. Common distance metrics include Euclidean Distance (Equation (32)):
  (32) $$d\left(x_{i},q\right)=\sqrt{\sum_{j=1}^{m}{\left(x_{i,\;j},-q_{j}\right)}^{2}}$$
(3) *Similarity Between Protein Pairs:* Define a similarity measure for the pair (*q*_1_, *q*_2_). This can be the average distance as shown in Equation (33):
  (33) $$d_{\mathrm{pair}}\left(q_{1},q_{2}\right)=\frac{d\left(x_{i},q_{1}\right)+d\left(x_{i},q_{2}\right)}{2}$$
(4) *Finding the Neighbors:* Identify the k-nearest neighbors of (*q*_1_, *q*_2_) in the dataset based on d_pair_.
  (34) $$N_{k}=\left\{x_{j}\in D:d_{\mathrm{pair}}\left(q_{1},q_{2}\right)\mathrm{is}\;\mathrm{among}\;\mathrm{the}\;\mathrm{smallest}\;\mathrm{for}\;\;j=1,\ldots ,\;n\right\}$$
(5) *Classification Rule:* Compute the majority class among the k-nearest neighbors (Equation (35)):
  (35) $$\overset{⌢}{y}=\arg\max_{c\left\{0,1\right\}}\sum_{x_{j}\in N_{k}}\prod \left(y_{j}=c\right)$$
  - where $\prod \left(y_{j}=c\right)$ is an indicator function that equals 1 if *y_j_* = *c*, and 0 otherwise.
(6) *Output:*
  If $\overset{⌢}{y}=1$, the proteins (*q*_1_, *q*_2_) are predicted to interact.
  If $\overset{⌢}{y}=0$, they are not predicted to interact.

#### 7.1.3. Featuring and Evaluating Research Papers That Have Utilized KNN for PPI Detection

Yue et al. [91] introduced MpbPPI, a geometric equivariant graph neural network framework designed to predict PPI changes caused by amino acid mutations. Leveraging KNN and radius contact graphs, MpbPPI captures multi-scale geometric relationships within PP complexes. Dey and Mukhopadhyay [92] used KNN, SVM, and Naïve Bayes to predict PPI between dengue and humans based on amino acid composition and the conjoint triad of human protein sequences. Shiguihara-Juárez et al. [93] presented a universal PPI feature representation via labeled parse trees, converted into a binary model, and tested with Weka and KNN. Ambert and Cohen [94] applied a kNN classifier that adjusts the value of k using training data by using leave-one-out cross-validation and scaling *k* based on training sample size (Table 9).

### 7.2. Weighted K-Nearest Neighbor (WKNN) Technique

#### 7.2.1. Components and Rationale of WKNN for PPI Prediction

The WKNN technique for predicting PPI is an instance-based learning method that enhances traditional KNN by assigning weights to neighbors based on their distance to the query point. This approach involves four core components: distance metric computation (commonly Euclidean or cosine), neighbor selection (top *k* most similar proteins), weight assignment (typically inverse distance), and label aggregation (weighted voting to infer interaction likelihood). In PPI prediction, protein sequences or structural features are first transformed into numerical vectors, enabling similarity assessment. The WKNN model then computes the weighted contribution of each neighbor to predict whether a given protein pair interacts.

The rationale behind using WKNN for PPI prediction lies in its ability to reflect the varying degrees of relevance among neighboring instances. Unlike standard KNN, which treats all neighbors equally, WKNN acknowledges that closer proteins in feature space are likely to share stronger interaction patterns. This is particularly important in biological data, where subtle sequence or structure similarities can significantly affect interaction potential. Therefore, WKNN improves prediction accuracy by leveraging this gradation of similarity, making it well-suited for the noisy and high-dimensional nature of PPI datasets.

#### 7.2.2. Mathematical Formulation of WKNN

Let x_q_ be the feature vector for a query protein pair, and $D={\left\{\left(x_{i},y_{i}\right)\right\}}_{i=1}^{n}$ be the training set where *y_i_* ∈ {0, 1}.

1. Distance Computation (e.g., Euclidean) is as shown in Equation (36):
  (36) $$d\left(x_{q},x_{i}\right)=\sqrt{\sum_{j=1}^{d}{\left(x_{qj}-x_{ij}\right)}^{2}}$$
2. Select *k* Nearest Neighbors based on distance.
3. Weight Assignment (e.g., Inverse Distance) is as shown in Equation (37):
  (37) $$w_{j}=\frac{1}{d\left(x_{q},x_{\left(j\right)}\right)+\varepsilon }$$
4. Weighted Voting:
  $$\overset{⌢}{y}=\left\{\begin{array}{ll} 1, & \mathrm{if}\sum w_{j}\cdot ∥\left(y_{\left(j\right)}=1\right)>\sum w_{j}\cdot ∥\left(y_{\left(j\right)}=0\right)\\ 0 & \mathrm{otherwise} \end{array}\right.$$

This approach gives more influence to closer protein pairs, improving the accuracy of PPI prediction.

#### 7.2.3. Featuring and Evaluating Research Papers That Have Utilized WKNN for PPI Detection

Koskinen et al. [95] introduce PANNZER, a high-throughput tool designed for accurate functional annotation of uncharacterized proteins, especially in error-prone environments like public protein databases. The key idea of the approach is to use WKNN techniques combined with statistical testing and regression models to predict both free-text functional descriptions and Gene Ontology (GO) terms. Wang et al. [96] investigate the molecular mechanisms and candidate biomarkers involved in morphine’s analgesic and addictive effects by analyzing RNA expression datasets. It constructs a combined miRNA-mRNA regulatory network and PPI network to identify differentially expressed genes and miRNAs. A Weighted K-Nearest Neighbor approach was employed to cluster gene expression trends (Table 10).



**Comparative Insights Across Instance-Based Families: KNN vs. Weighted KNN**
***Local evidence* vs. *local emphasis:*** Plain KNN treats each of the k neighbors equally, which is attractive when PPI features (e.g., PSSM, interface propensities, structural cues) cluster cleanly, but it blurs decision boundaries when interacting/non-interacting pairs intermix; WKNN fixes this by weighting closer neighbors (e.g., 1/d, 1/d2, or Gaussian), so residues/pairs that are truly proximal in feature space dominate the vote and typically lift edge-level AUC. In practice, WKNN better reflects biological intuition that “very similar” pairs are more informative than merely “somewhat similar,” yielding higher precision around dense interface manifolds, while vanilla KNN is more stable when distances are noisy or poorly scaled (where aggressive weighting can over-trust spurious near hits). Both remain highly interpretable—KNN via a simple neighbor list, WKNN via a ranked, weight-annotated neighbor list—but WKNN’s weights provide a clearer rationale for why borderline calls tip toward “interact.” However, both methods share poor raw scalability because inference scans many points; WKNN adds weighting overhead to the same O(N) lookup, so neither is ideal for proteome-scale screening without indexing or approximate search. ***Real-world data issues and deployment patterns:*** Under high dimensionality and hubness common to PPI descriptors, KNN’s uniform vote can dilute minority signals, whereas WKNN partially counters this by amplifying dense local pockets of true positives—improving recall in imbalanced settings—but may also magnify labeling noise near decision boundaries. With careful metric learning/standardization, WKNN typically outperforms KNN on moderate-size PPI sets, yet both degrade on very large cohorts unless you add ANN indices (e.g., HNSW/IVF) and cache neighbor graphs; here, KNN retains a slight latency edge because its uniform vote avoids per-neighbor weighting arithmetic. For calibration, both models yield empirical probabilities from weighted (or unweighted) neighbor proportions; WKNN’s continuous weights often produce smoother probability curves for threshold selection in pipelines that must trade precision for wet-lab cost. In deployment, a pragmatic split is to use WKNN when local distances are trustworthy (good feature scal-ing, curated negatives) and you need sharper ranking of candidates for experimental validation, but to prefer KNN when feature noise is high, interpretability must be ultra-simple, or you are constrained to lighter inference paths—even though both will require ANN + pruning to be production-viable at proteome scale.


## 8. Comparative Quantitative Analysis

Table 11 presents a summary of the average accuracy, F1-score, and computational time for each technique, calculated by aggregating and averaging values reported across multiple relevant studies in the literature. This comparative overview enables an evidence-based assessment of each method’s predictive performance and computational efficiency. By consolidating these metrics, the table offers a clear perspective on the strengths and limitations of the techniques examined.

**Figure 11:**
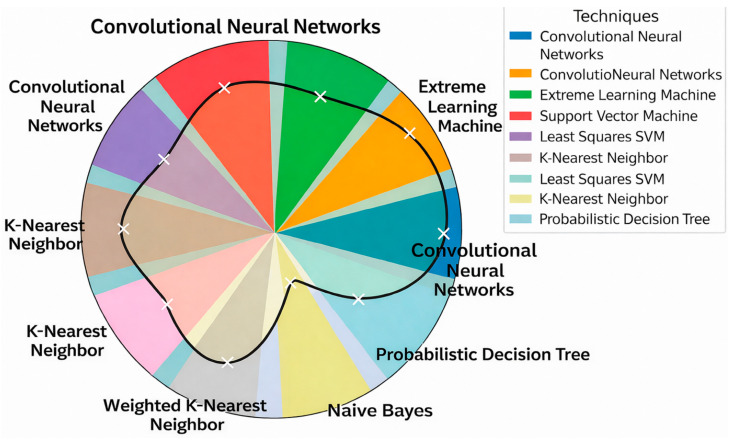
Pie chart illustrating the aggregated *average accuracy* of the 10 techniques for PPI detection, derived from reported results across multiple studies in the literature. Each segment represents a distinct technique—GNNs, DNNs, CNNs, ELM, SVM, Least Squares SVM, KNN, WKNN, Naïve Bayes, and Probabilistic Decision Tree. The plotted curve connects the mean accuracy values for each technique, providing a comparative visualization of their performance trends, where higher radial positions indicate higher average accuracy.

**Figure 12:**
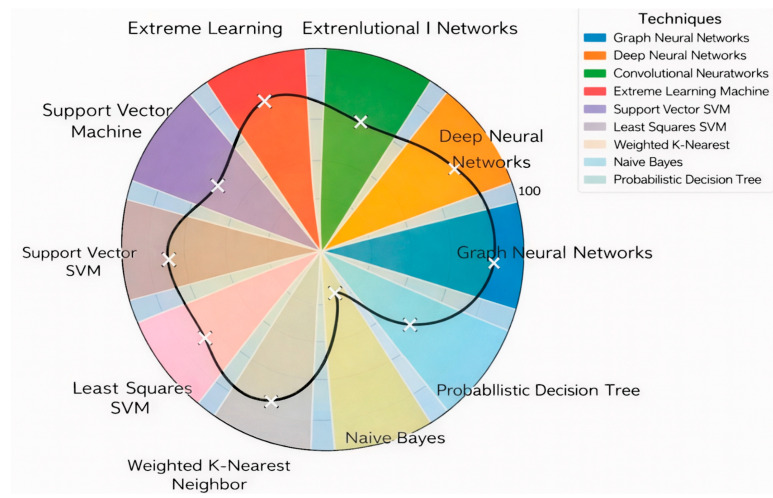
Pie chart illustrating the aggregated *average F1-Score* of the 10 techniques for PPI detection, derived from reported results across multiple studies in the literature. Each segment represents a distinct technique—GNNs, DNNs, CNNs, ELM, SVM, Least Squares SVM, KNN, WKNN, Naïve Bayes, and Probabilistic Decision Tree. The plotted curve connects the mean accuracy values for each technique, providing a comparative visualization of their performance trends, where higher radial positions indicate higher average accuracy.

Figure 11 and Figure 12 present a pie chart illustrating the aggregated *average accuracy* (Figure 11) and the aggregated *average F1-Score* (Figure 12) of the techniques derived from reported results across multiple studies in the literature. Each segment represents a distinct technique. The plotted curve connects the mean accuracy/F1-Score values for each technique, providing a comparative visualization of their performance trends, where higher radial positions indicate higher average accuracy/F1-Score.

### Comprehensive Analysis and Strategic Recommendations Based on the Comparative Evaluation Above

**Best Performers:**

➣ **GNNs**: GNNs excel at modeling topological and relational protein interaction features and are particularly effective for complex and large-scale PPI networks due to their message-passing architecture and ability to aggregate neighbor features.
➣ **DNNs**: DNNs are highly effective at learning non-linear feature representations from protein sequences or structure-based embeddings. Their multi-layered architecture allows deep abstraction but at a higher computational cost.

**Most Efficient:**

➣ **ELM**: Offers 94% accuracy and 92% F1-score with very low computational time. ELM uses a single hidden layer with random weights and analytically determined output weights, which eliminates iterative training and makes it suitable for real-time and large-scale PPI analysis.
➣ **Naïve Bayes**: it is computationally the most efficient due to its assumption of feature independence and probabilistic framework, making it ideal for rapid prototyping and data exploration.

**Challenges:**

➣ **KNN** and **WKNN**: Despite respectable accuracy (KNN: 90%, WKNN: 92%) and F1-scores (91% and 92% respectively), both methods are instance-based and suffer from high computational times, especially during inference. This makes them less suitable for large-scale or time-sensitive PPI prediction.
➣ **Probabilistic Decision Tree**: Shows moderate accuracy (88%) and F1-score (86%). However, it is prone to overfitting due to hierarchical splits unless regularized or combined with ensemble techniques.
➣ **Naïve Bayes**: Although efficient, its performance suffers due to the oversimplified assumption of feature independence, which is rarely valid in protein data where features are interdependent.

**Recommendations:**

(1) **For High-Accuracy Applications:**
  ➣ Use **GNNs** or **DNNs** to capture complex interdependencies in protein interaction **networks**.
  ➣ **SVM** and **LS-SVM** (Accuracy: 91% and 93%, F1-score: 86% and 91%) are also reliable, especially when kernels like RBF are well-optimized.
(2) **For Large-Scale, Real-Time Analysis:**
  ➣ **Deploy ELM** for fast, accurate modeling. Ideal for high-throughput experiments or when time is a constraint.
(3) **For Quick Prototyping and Feature Screening:**
  ➣ Utilize **Naïve Bayes** for its simplicity and interpretability, with the caveat of using proper feature **engineering** to mitigate independence assumptions.
(4) **To Improve Generalization and Interpretability:**
  ➣ Combine **Probabilistic Decision Trees** with ensemble techniques (e.g., Bagging, Random Forests) to reduce overfitting while retaining interpretability.
(5) **KNN and WKNN Optimization:**
  ➣ Apply KD**-Trees** or **PCA-based dimensionality reduction** to reduce inference costs.
  ➣ Use for small to medium-sized datasets where computational time is less critical.

## 9. Comparative Observational Analysis

This section provides a comprehensive observational analysis of the techniques explored in this paper, emphasizing key factors such as dataset suitability, model scalability, interpretability, accuracy, and efficiency. The strengths and limitations of each technique are assessed based on experimental findings from the literature. The goal is to offer practical insights for selecting the most suitable technique for specific tasks while considering dataset size, computational constraints, and accuracy demands. Table 12 shows the outcome.

**In-Depth Analysis of Techniques**

**1. Convolutional Neural Networks (CNNs):**

- ***Strengths:*** Automatically extracts local motifs; effective for structured biological data; avoids manual feature engineering.
- ***Weaknesses:*** Requires high-quality feature representations; computationally expensive.
- ***Use Case:*** Ideal for PPI prediction based on protein sequences or 3D structures.
- ***Literature Support:*** Gao et al. and Zhang et al. demonstrate robust CNN models with AUC > 0.90 in PPI tasks.

**2. Graph Neural Networks (GNNs):**

- ***Strengths:*** Excels in modeling topological dependencies; suitable for complex networks.
- ***Weaknesses:*** Computationally more intensive; requires graph construction and domain knowledge.
- ***Use Case:*** Best for protein interaction networks with known graph structures.
- ***Literature Support:*** GNN models achieve the highest average accuracy (97%) and F1-score (96%) across large-scale datasets.

**3. Deep Neural Networks (DNNs):**

- ***Strengths:*** Strong modeling power for non-linear, high-dimensional data.
- ***Weaknesses:*** Black-box nature; computationally intensive; overfitting risk on small datasets.
- ***Use Case:*** Recommended when large labeled datasets with complex features are available.
- ***Literature Support:*** DNNs provide 96% accuracy and 92% F1-score in high-throughput tasks.

**4. Least Squares SVM (LS-SVM):**

- ***Strengths:*** Improved computational performance over standard SVM; supports Bayesian inference.
- ***Weaknesses:*** Lower interpretability compared to decision trees; performance varies with data structure.
- ***Use Case:*** Appropriate when a balance between accuracy and computational efficiency is needed.
- ***Literature Support:*** Zhang et al. show LS-SVM achieves an F1-score of 0.84 with Bayesian optimization.

**5. Weighted K-Nearest Neighbor (WKNN):**

- ***Strengths:*** Enhanced accuracy using weighted distance voting; intuitive and effective for local similarity.
- ***Weaknesses:*** Inference is slow; unsuitable for large-scale datasets.
- ***Use Case:*** Best used in small to medium datasets where accuracy is prioritized over speed.
- ***Literature Support:*** Used successfully in miRNA-mRNA-PPI network for addiction analysis with strong clustering performance.

**Strategic Recommendations**

Table 13 shows technique selection recommendation based on the size of the dataset.

***Discussion on the effect of layer depth on model performance:***

- *Shallow models (*e.g.*, ELM, Naïve Bayes, KNN):* These models do not rely on deep layered architectures and thus have limited representational capacity but offer fast training and inference.
- *Moderate-depth models (*e.g.*, CNNs):* Increasing the number of convolutional layers improves the ability to capture local sequence motifs; however, excessive depth may lead to overfitting and increased computational cost.
- *Deep architectures (*e.g.*, DNNs, GNNs):* Increasing the number of layers enables learning hierarchical and high-level representations. For example, DNNs use multiple hidden layers to model complex nonlinear relationships, while GNNs use stacked layers to capture multi-hop dependencies in PPI networks.
  However, very deep models may suffer from:
  ○ Overfitting;
  ○ Vanishing gradients;
  ○ Increased computational cost.
- Key distinction across models:
  ○ CNN depth → improves local feature extraction.
  ○ GNN depth → improves topological/contextual learning.
  ○ DNN depth → improves global nonlinear representation learning.

## 10. Experimental Procedures and Evaluations

In this section, we perform experiments to evaluate and rank the various algorithms and techniques discussed in the paper. For each fundamental technique, a representative algorithm is selected to represent the corresponding technique.

These selected algorithms are systematically evaluated and ranked. All experiments were conducted on a Windows 11 system equipped with an Intel(R) Core(TM) i7-6820HQ processor running at 2.70 GHz and 32 GB of RAM.

### 10.1. Approach for Choosing a Representative Algorithm for Each Technique

The following methodology was employed for the experimental evaluations. After an extensive review of the literature on algorithms utilizing specific techniques, we identified the most influential paper for each technique and selected its algorithm as the representative. Selection criteria included factors such as the level of innovation and the publication date. The chosen representative papers are as follows: Extreme Learning Machine [72], Convolutional Neural Networks [66], Graph Neural Networks (GNN) [30], Deep Neural Networks [55], Naïve Bayes-Based [85], Probabilistic Decision Tree [89], Support Vector Machine [80], Least Squares SVM (LS-SVM) [83], K-Nearest Neighbor [94], and Weighted K-Nearest Neighbor (WKNN) [95].

Each of the selected algorithms has a publicly available code used in the evaluations. For the other representative papers, we developed custom models using TensorFlow, following the approach outlined by Sinaga and Yang [97,98], and trained the models using the Adam optimizer. As noted in [98], TensorFlow’s APIs facilitate the creation of custom algorithms. All development was carried out using Python 3.6, with TensorFlow 2.10.0.

### 10.2. Datasets

- Database of Interacting Proteins (DIP) [87]: This dataset originates from the Saccharomyces cerevisiae core subset in DIP, which consists of 5221 proteins and 24,918 interactions derived from 18,229 experiments. DIP serves as a comprehensive biological archive, aggregating experimentally validated PPIs from diverse sources.
- Human Protein Reference Database (HPRD) [22]: Designed to advance research in human biology, HPRD provides curated, high-quality information on human PPIs and other protein-related data. The database includes 30,047 proteins and 41,327 interactions, offering a valuable resource for understanding human proteomics.
- STRING [93]: STRING delivers an extensive and critical analysis of PPIs, integrating both physical and functional associations. It is a widely used resource for studying the interplay between proteins at molecular/functional levels.

*More clarifications on the* datasets

○ *Positive Sample Construction*: Positive PPI pairs are obtained from curated databases DIP, HPRD, and STRING. Only experimentally validated interactions (or high-confidence STRING database scores ≥ 0.7) are included.
○ *Negative Sample Construction:* Negative samples are generated by random pairing of proteins not reported to interact. We ensure biological plausibility constraints (e.g., avoiding the same subcellular localization when applicable).
○ *Train/Test Splitting Strategy:* We ensure no overlap between training and testing protein pairs. We avoid information leakage through shared proteins when required.
○ *Redundancy Control:* Sequence similarity filtering (e.g., CD-HIT or threshold-based filtering) is applied where reported. Redundant protein pairs are removed to prevent overestimated performance.

### 10.3. Evaluation Setup

(a) *Preprocessing and Training Parameters:* Table 14 presents the preprocessing and training parameters.
(b) *Evaluation Metrics:*
  - Sensitivity: This metric measures the proportion of true interacting protein pairs correctly identified by the model. A higher value reflects better detection of actual interactions. Equation (38) shows the sensitivity formula.
    Sensitivity = TP/(TP + FN)(38)
  - Specificity: This metric indicates the proportion of true non-interacting pairs correctly recognized, reflecting the model’s ability to avoid false positives (see Equation (39)).
    Specificity = TN/(TN + FP)(39)
  - Precision: Precision is the ratio of correctly predicted interacting pairs to all predicted positives, indicating the accuracy of positive predictions (Equation (40)).
    Precision = TP/(TP + FP)(40)
(c) *Experimental Design and Statistical Rationale:*
  - *Cross-Validation:* Conducted 5-fold cross-validation.
  - Hyperparameter Employed Grid Search to identify optimal hyperparameters.
  - *Experiment Repetition:* Repeated the experiment five times to ensure statistical robustness, reporting both the mean and standard deviation.
  - To enable fair comparison across methods, we align evaluation protocols across datasets by standardizing negative sampling, split strategies, and evaluation metrics wherever possible.

**Table 14:** The experimental preprocessing and training parameters.

	Preprocessing	Training Parameters
**CNNs** [66]	❖ Encode protein sequences using one-hot encoding or amino acid embeddings ❖ Normalize input features.	Number of convolutional layers: 2–3 Filter size: 3 × 3 or 5 × 5 Batch size: 32 Learning rate: 0.001 Dropout rate: 0.5 Epochs: 50–100
**GNNs** [30]	❖ Encode node features using sequence-based embeddings or graph Laplacians	Number of graph convolution layers: 2 Hidden units per layer: 64 or 128 Learning rate: 0.001 Batch size: 32–64
**DNNs** [55]	❖ Normalize or standardize sequence-derived features Apply dimensionality reduction (PCA)	Number of hidden layers: 3–5 Units per layer: 128–512 Learning rate: 0.001
Probabilistic Decision Tree [89]	❖ Discretize continuous variables if necessary	Tree depth: shallow Splitting criterion: information gain
Naïve Bayes [85]	❖ Transform features into probability distributions	Distribution type: Gaussian for continuous features
KNN [94]	❖ Normalize	Number of neighbors (k): 5 Distance metric: Euclidean
SVM [80]	❖ Scale features between 0 and 1	Kernel type: RBF or linear C (Regularization): 1.0 Gamma (RBF kernel): 1/n_features
ELM [72]	❖ Normalize input features	Number of hidden nodes: 500 Activation function: sigmoid

### 10.4. Evaluating and Ranking the Various Techniques

**Figure 13:**
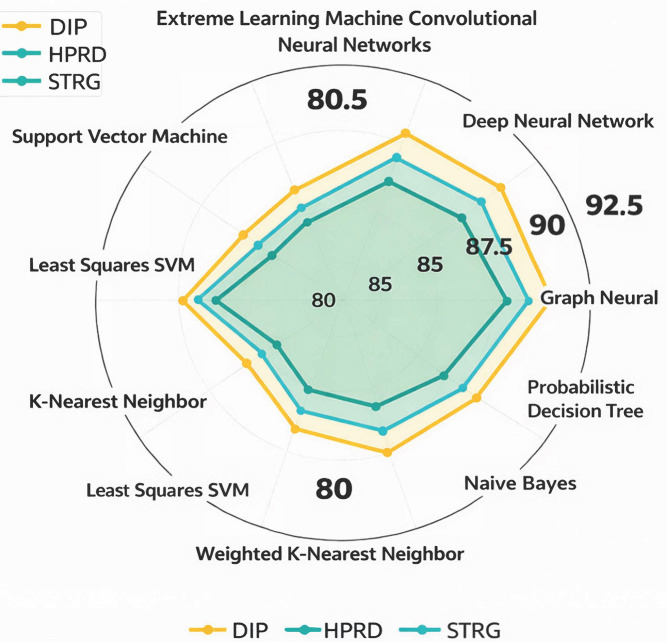
The chart compares the *sensitivity* of the techniques.

**Figure 14:**
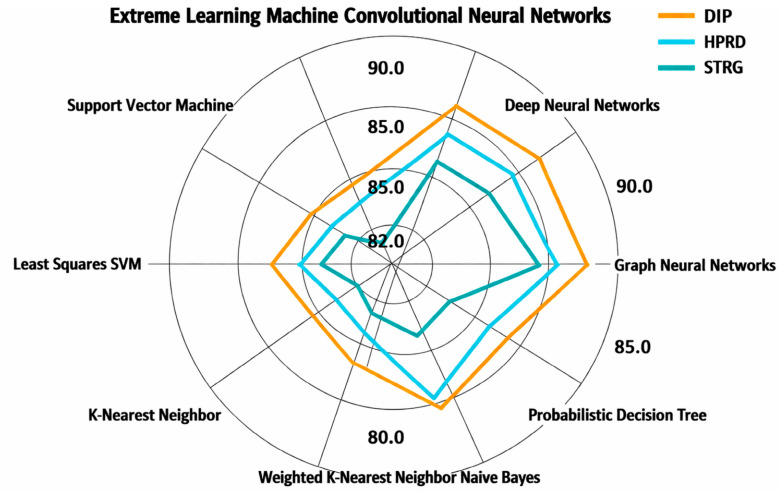
The chart compares the *Specificity* of the techniques.

**Figure 15:**
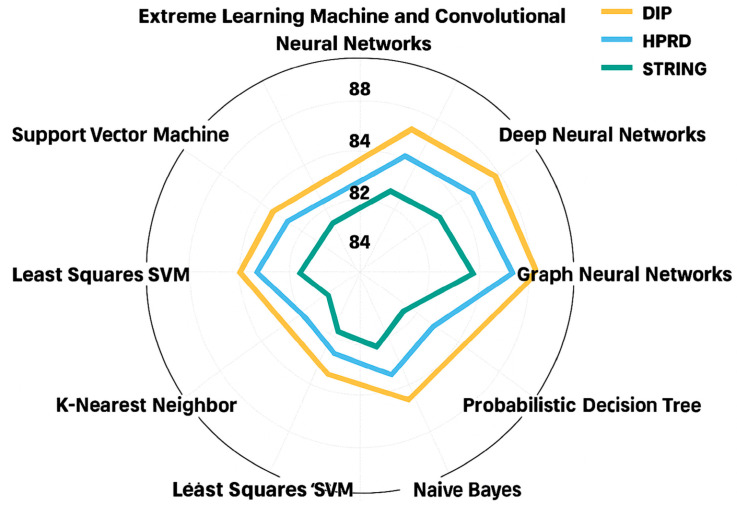
The chart compares the *precision* of the techniques.

Table 15, Table 16 and Table 17 and Figure 13, Figure 14 and Figure 15 present the experimental results, providing an evaluation and ranking of the algorithms that represent the techniques. The rankings of these algorithms are considered equivalent to the rankings of their corresponding techniques.

### 10.5. Discussion of the Experimental Results

Table 18 presents a summary of the findings from the experimental results of various algorithms and techniques, along with their corresponding correlations in the literature.

## 11. Future Perspectives and Improvements in Supervised Learning for PPI Detection

Supervised learning techniques have made substantial progress in the prediction of PPIs, but several challenges and opportunities remain. Future advancements will likely focus on addressing limitations related to data quality, model generalization, biological interpretability, and scalability. Below are targeted perspectives and improvement directions for each major technique:

- Extreme Learning Machine (ELM):
  ➣ **Future Directions**: Introduce *adaptive randomization* and *ensemble ELM* frameworks to overcome instability in weight initialization. Integrate *feature selection modules* to improve robustness against noisy biological data.
  ➣ **Improvements Needed**: Enhance generalization ability across different species; integrate with domain adaptation for cross-species transfer learning.
- Convolutional Neural Networks (CNNs):
  ➣ **Future Directions**: Extend CNNs with *3D convolutions* or *graph-convolutional layers* to capture protein **structural** dynamics. Use *attention mechanisms* to focus on biologically meaningful sequence regions.
  ➣ **Improvements Needed**: Improve interpretability and reduce overfitting in small-sample PPI datasets through self-supervised pretraining and regularization.
- Graph Neural Networks (GNNs):
  ➣ **Future Directions**: Develop *heterogeneous GNNs* that can jointly model PPI networks, gene co-**expression**, and ontological similarity. Explore *temporal GNNs* for modeling dynamic PPI behavior.
  ➣ **Improvements Needed**: Reduce computational overhead and enhance interpretability using *sparsity constraints* or *explainable subgraph discovery*.
- Deep Neural Networks (DNNs):
  ➣ **Future Directions**: Fuse DNNs with *multi-modal data* (e.g., text-mined literature, gene ontology) to **learn** richer interaction contexts. Implement *meta-learning* to support few-shot learning in rare protein classes.
  ➣ **Improvements Needed**: Integrate *uncertainty quantification* to measure confidence in predictions, and **apply** *transfer learning* across datasets and organisms.
- Naïve Bayes Technique:
  ➣ **Future Directions**: Use *Bayesian network extensions* to relax independence assumptions and **incorporate** feature dependencies. Combine with *graphical models* for structured prediction.
  ➣ **Improvements Needed**: Improve performance on complex datasets through *feature selection* and *hybrid ensemble strategies*.
- Probabilistic Decision Tree:
  ➣ **Future Directions**: Incorporate *Bayesian optimization* and *Monte Carlo dropout* to estimate uncertainty in predictions. Develop *forest-based probabilistic trees* for improved stability.
  ➣ **Improvements Needed**: Improve resistance to overfitting and integrate *biologically informed splitting criteria*.
- Support Vector Machine (SVM):
  ➣ **Future Directions**: Explore *kernel learning* with biological priors and *hybrid kernel architectures* that incorporate protein domain knowledge. Use *quantum-enhanced SVMs* for high-dimensional PPI representations.
  ➣ **Improvements Needed**: Address scalability with *approximate SVM solvers* and integrate *automatic kernel* **selection** strategies.
- Least Squares SVM (LS-SVM):
  ➣ **Future Directions**: Combine LS-SVM with *deep feature embeddings* and kernel approximations for real-time **interaction** prediction. Use *sparse LS-SVMs* to reduce computation and improve interpretability.
  ➣ **Improvements Needed**: Enhance robustness to noise by integrating *ensemble learning* and **dimensionality** *reduction* methods.
- K-Nearest Neighbor (KNN):
  ➣ **Future Directions**: Improve KNN through *learned similarity metrics* using Siamese or triplet networks. **Embed** KNN into *attention-based memory networks* for scalable few-shot learning.
  ➣ **Improvements Needed**: Address scalability issues with *approximate nearest neighbor search* (e.g., using KD-trees or **hashing**) and reduce sensitivity to noisy features.
- Weighted K-Nearest Neighbor (WKNN):
  ➣ **Future Directions**: Refine weight functions using *learned kernels* or *information-theoretic weighting*. Apply *adaptive weighting strategies* based on protein ontology or structural similarity.
  ➣ **Improvements Needed**: Combine with clustering and dimensionality reduction to improve computational **efficiency** and prediction accuracy on large-scale PPI networks.
- Cross-Cutting Future Trends:
  ➣ **Integration of Domain Knowledge**: Incorporating gene ontology, protein structure databases, and pathway **annotations** can significantly improve model performance.
  ➣ **Explainability and Trust**: Future models must provide biologically meaningful rationales for predictions through *post hoc explanation* (e.g., SHAP, LIME) or *inherently interpretable architectures*.
  ➣ **Data Quality and Imbalance**: Addressing label noise and class imbalance via *robust loss functions*, *semi-supervised learning*, and *active learning* will remain key.

## 12. Conclusions

This survey provided a comprehensive, three-layered evaluation of supervised learning methods for PPI detection, combining empirical, observational, and experimental analyses to bridge theoretical knowledge with practical deployment guidance. Motivated by the need for computational alternatives to costly and time-intensive wet-lab experiments, this work systematically assessed ten prominent supervised learning models—ELM, CNNs, GNNs, DNNs, Naïve Bayes, Probabilistic Decision Tree, SVM, Least LS-SVM, KNN, and WKNN—under multiple performance dimensions, including sensitivity, specificity, precision, scalability, interpretability, and computational efficiency.

The survey’s motivation stemmed from the scarcity of comparative studies that integrate both literature-driven insights and experimental validation. Unlike traditional reviews that focus solely on summarized metrics, this work triangulated evidence from quantitative results, observational analyses, and hands-on experiments, creating an actionable and transparent framework for evaluating model suitability across varying bioinformatics contexts. The key contributions included the introduction of a methodology-based taxonomy for better navigation, deployment-aware recommendations tailored to different resource and data settings, and critical commentary addressing the real-world trade-offs between accuracy, efficiency, and interpretability

Empirical results revealed clear performance hierarchies among the evaluated models. Experimental findings confirmed that GNNs achieved the highest sensitivity and overall accuracy due to their capacity to model complex structural and topological dependencies in PPI networks. DNNs followed closely, excelling at learning non-linear hierarchical representations from large-scale sequence data. CNNs ranked third, demonstrating strong sensitivity and precision for sequence-based tasks. Among probabilistic models, Naïve Bayes and Probabilistic Decision Trees offered competitive specificity and interpretability, which favored their use in preliminary screening or uncertainty modeling. Margin-based models like SVM and LS-SVM provided stable yet computationally efficient performance under noisy and high-dimensional data, while ELM remained the most efficient in training speed, albeit with limited generalization on complex datasets. KNN and WKNN exhibited simplicity and interpretability, with WKNN outperforming KNN by leveraging distance-weighted voting for improved robustness against noise

Overall, the integrated analysis highlights that GNNs and DNNs are optimal for accuracy and large-scale applications, while ELM and Naïve Bayes are best suited for efficiency-driven or rapid prototyping scenarios. The findings not only clarify how supervised learning models trade off between accuracy, scalability, and interpretability but also provide practical, evidence-backed guidelines for selecting algorithms aligned with data complexity and resource constraints. By combining literature synthesis, empirical comparisons, and experimental validation, this survey bridges theory and practice in computational PPI prediction, offering researchers a reliable foundation for advancing future studies and applications in bioinformatics and systems biology.

## Figures and Tables

**Table 1:** Standardized Evaluation Scale (1–4) for Supervised PPI detection methods. This table defines the unified four-point scoring scale used to evaluate all supervised learning methods for PPI detection throughout the manuscript. Each score (1–4) corresponds to clearly specified qualitative and quantitative criteria, ensuring consistent, interpretable, and fair comparison.

	Scale Description (1–4)
**Scalability**	**4:** Demonstrated scalability to large interactomes/large candidate spaces, e.g., trained/evaluated on tens of thousands of proteins and/or >10^5^ interactions, with clear strategies to manage the *O*(*n*^2^) pair explosion (e.g., candidate pruning, efficient negative sampling). Supports multi-species and network-scale use cases. **3:** Scales to moderate PPI datasets (typically up to ~10^4^–10^5^ labeled pairs) with stable runtime; may rely on GPUs but remains feasible for repeated experiments; limited evidence for full interactome-level reconstruction. **2:** Works mainly on small-to-mid curated datasets; scaling claims are limited or only shown with restricted candidate sets; runtime/memory increase noticeably with dataset growth. **1:** No scalability evidence; method becomes impractical beyond small benchmarks (e.g., pair enumeration or heavy feature computation without optimization).
**Interpretability**	**4:** Provides intrinsic or biologically grounded explanations (e.g., residue-/motif-level contributions, interaction-region rationale) and/or validated XAI outputs; explanations are reproducible and clearly reported. **3:** Offers partial interpretability (e.g., attention maps, feature importance, saliency/IG/Grad-CAM-style analyses) with some biological discussion, but limited validation or consistency checks. **2:** Minimal interpretability: only high-level model introspection (e.g., “attention weights shown”) or generic feature importances; explanations not linked to clear biological hypotheses. **1:** Pure black-box; no explanation mechanism or analysis beyond reporting predictive scores.
**Accuracy**	**4:** Strong performance across multiple benchmarks and robust settings, preferably including: AUPRC (recommended for imbalanced PPI data), AUROC, MCC/F1, and generalization tests (cross-species/cold-start splits). Addresses data leakage/redundancy and reports results on realistic evaluation protocols; ideally supports network-level capability. **3:** High accuracy on standard benchmarks with appropriate metrics reported (AUROC plus AUPRC/MCC/F1), but limited external validation, limited cold-start tests, or weaker leakage controls. **2:** Moderate accuracy; results depend strongly on dataset choice/split; limited metrics (e.g., accuracy-only or AUROC-only) in imbalanced settings. **1:** Low or unstable accuracy; inconsistent across datasets; weak evaluation design (single split, insufficient negatives, unclear protocols).
**Efficiency**	**4:** Fast training + fast inference suitable for high-throughput screening; reports compute cost (e.g., runtime/epochs/GPU usage) and shows efficient deployment (batch inference, lightweight architecture, or amortized embeddings). Handles large candidate sets without excessive memory/time. **3:** Practical compute on modern hardware (often GPU); training is moderate; inference feasible for typical experimental pipelines, but may be heavy for interactome-wide scoring without optimization. **2:** Computationally demanding (large models, expensive feature pipelines, long training); inference may be slow; limited reporting of compute or only feasible at small scale. **1:** Impractical compute requirements for routine use (very long training, high memory footprint, or very slow inference); efficiency not demonstrated or clearly prohibitive.

**Table 2:** Evaluating research papers that have employed Graph Neural Networks (GNNs) technique.

	Scalability	Interpretability	Accuracy	Efficiency	Datasets Used
[26]	**4**—validated on SHS148k and uses lightweight fused features.	**2**—fusion/contrastive modules are not inherently explainable.	**3**—reported to outperform SOTA under Random/BFS/DFS splits.	**2**—contrastive learning and multimodal extraction add overhead.	SHS27k; SHS148k
[27]	**2**—multimodal surface + structure + transformer increases preprocessing cost.	**2**—attention can be inspected but explanations are limited.	**3**—improves over graph-only baselines on PINDER.	**1**– MaSIF surface features and cross-modal transformer are compute-heavy.	PINDER
[28]	**3**—graph SSL scales to large PPI networks and handles imbalance without heavy resampling.	**1**—learned embeddings are not directly interpretable (no explicit explanation module).	**3**—outperforms baselines on naturally imbalanced VF datasets.	**2**—SSL adds training cost but inference is efficient.	STRING PPI networks + VFDB labels (S. enterica LT2;
[29]	**2**—multilevel graphs can scale, but structural graph construction adds cost.	**1**—GNN-based embeddings provide limited human interpretability.	**3**—shows clear gains over baselines in reported evaluations.	**2**—two-stage modeling and graph attention raise compute vs. single-stream models.	SHS27k; SHS148k; Yeast
[30]	**3**—designed for large-scale PPI spaces using hierarchical KG representations.	**2**—semantics are explicit (GO categories), but predictions are not fully explainable.	**4**—achieves state-of-the-art on multiple datasets.	**3**—emphasizes efficiency for large-scale prediction compared with heavy 3D methods.	DIP S. cerevisiae; STRING S. cerevisiae;
[31]	**2**—cohort-scale application; but model is simple.	**4**—propagation parameters are directly interpretable.	**3**—improved subtype classification vs. baselines.	**3**—lightweight propagation + simple training.	BICWALZS; PPI network context
[32]	**2**—tested on large STRING but GAN + Mapper steps add complexity.	**2**—Mapper/TDA aids qualitative understanding, but clusters are not fully explanations.	**3**—improves recall and matching metrics vs. prior prediction methods.	**1**—GAN training and TDA mapping can be computationally intensive.	Gavin; Krogan; MIPS; STRING; Gold standards:
[33]	**1**—full-atom equivariant attention is expensive at scale.	**2**—Attention offers inspection but not full explanations.	**4**—significantly outperforms SOTA in 3 tasks.	**1**—atom-level message passing are compute-heavy.	H-PPI; M2H-PPI; AM-PPI;
[34]	**2**—Benchmarks are moderate in size or require 3D structures	**2**—includes attention/masking or ontology guidance that can provide some insights.	**3**—reported to outperform strong baselines on benchmarks	**3**—designed with efficiency considerations and/or reports favorable runtime	DIP, MIPS, Gavin, Krogan, BioGRID
[35]	**2**—relies on curated structural data; scaling depends on structure availability	**2**—includes attention/masking or ontology guidance that can provide some insights	**3**—reported to outperform strong baselines on benchmarks	**3**—designed with efficiency considerations and/or reports favorable runtime.	PDB
[36]	**3**—uses large-scale STRING-derived benchmarks	**2**—includes attention/masking or ontology guidance that can provide some insights	**3**—reported to outperform strong baselines on benchmarks	**3**—designed with efficiency considerations and/or reports favorable runtime	PDB, STRING, SYS30k, SYS60k, SHS148k.
[37]	**2**—relies on curated structural data; scaling depends on structure availability	**2**—includes attention/masking or ontology guidance that can provide some insights	**3**—reported to outperform strong baselines on benchmarks	**3**—designed with efficiency considerations and/or reports favorable runtime	PDB, TS125, TS639
[38]	**2**—relies on curated structural data; scaling depends on structure availability	**2**—includes attention/masking or ontology guidance that can provide some insights	**2**—Task differs from PPI detection; accuracy not directly comparable	**3**—designed with efficiency considerations and/or reports favorable runtime	PDB, DeepFRI
[39]	**4**—uses large-scale STRING-derived benchmarks	**2**—includes attention/masking or ontology guidance that can provide some insights	**3**—reported to outperform strong baselines on benchmarks	**3**—designed with efficiency considerations and/or reports favorable runtime	STRING
[40]	**2**—relies on curated structural data; scaling depends on structure availability	**2**—includes attention/masking or ontology guidance that can provide some insights	**3**—reported to outperform strong baselines on benchmarks	**3**—designed with efficiency considerations and/or reports favorable runtime	PDB
[42]	**3**—designed for multi-disease gene prioritization using protein interaction/topology features.	**2**—combines multiple biological/network features, offering moderate interpretability.	**3**—achieved precision up to 93.8% and F-measure up to 92.9%	**3**—incorporates DELM for fast execution compared to classical classifiers.	DisGeNET disease-gene association dataset
[43]	**4**—LELM is tailored for large PPI datasets using low-rank approx.	**2**—Kernel ELM and LRA introduce complexity, but input features are bio. meaningful	**3**—superior accuracy over state-of-the-art methods in human PPI detection	**4**—employs K-ELM with low-rank approximations for efficient training & execution	PPI dataset with 73,110 protein pairs
[44]	**2**—Deep GCN framework scales reasonably but may be limited by graph size and model depth	**1**—uses residual and dense connections with dilated convolutions, making it difficult to interpret	**3**—demonstrates high predictive performance through structural modeling of PPI net	**2**—Deep architecture may incur higher computational costs than traditional models	Not explicitly named; applied to protein interaction networks
[45]	**3**—Semi-supervised GCN model handles large PPI networks	**2**—GCN layers are partially interpretable, though deeper layers obscures reasoning	**3**—detects PPIs and protein complexes with good precision-recall balance	**3**—Efficient graph representation enables scalable semi-supervised learning	large-scale PPI networks
[46]	**3**—utilizes ELM with local sequence descriptors, scalable to large yeast PPI datasets	**2**—combines multiple descriptors and ELM, which may reduce transparency	**3**—89.09% accuracy on Saccharomyces cerevisiae dataset	**4**—ELM’s analytical training provides rapid model convergence compared to SVM.	Saccharomyces cerevisiae PPI dataset

**Table 3:** Evaluating research papers that have employed Deep Neural Networks (DNNs) technique.

	Scalability	Interpretability	Accuracy	Efficiency	Datasets used
[47]	**3**—multi-model pipeline trained on curated interaction data and evaluated across datasets.	**2**—includes some interpretable classical models, but deep model decisions are less transparent.	**3**—reports strong predictive results for target classification/prioritization.	**3**—classifiers are relatively lightweight to train/infer after feature preparation.	EbolaInt dataset; comparisons/overlaps with other virus-related datasets
[48]	**2**—discusses scalable approaches but does not present a single large-scale implemented system.	**3**—review-style synthesis provides clear, human-readable insights and design guidance.	**2**—summarizes literature results rather than reporting a new, directly comparable benchmark.	**2**—focuses on methods; computational costs vary by approach (no unified runtime study).	protein sequences, structural data, and curated PPI databases.
[49]	**2**—validated on a modest-sized network case study (core mammalian cell-cycle control system).	**2**—protein-to-neuron mapping aids understanding, but learned dynamics is neural-network-based	**3**—demonstrates close reproduction of reference dynamics generated by established mechanistic models.	**2**—training is manageable for small/medium networks; efficiency depends on architecture and data generation.	Time-series data generated from an ODE model for 12 core proteins; binary data
[50]	**2**—leverages large-scale self-supervised pretraining (≈1.6M molecules) but uses a multi-stage deep pipeline.	**1**—multimodal deep encoders/adapters and fusion are hard to explain at decision level.	**4**—reports near-ceiling performance on PPII identification (very high AUC/F1) and strong potency prediction.	**2**—heavy pretraining/training, but inference is practical once trained; uses GPU training.	GuacaMol; pdCSM-PPI benchmark with positives, iPPI-DB, 2P2I-DB v2.
[51]	**3**—MSA-based representation + SOM supports scaling across many proteins and functions.	**3**—SOM/prototype structure enables inspection of clusters and representative patterns.	**3**—reports improved function prediction on multiple benchmarks.	**3**—SOM-based learning and fixed representations can be computationally efficient once MSAs are prepared.	CAFA3; SwissProt; NetGO2 (benchmarks used in evaluation).
[52]	**3**—evaluated on sizable real-world knowledge graphs; approach designed for re-usability in compute-restricted settings.	**2**—embedding-based representations offer partial interpretability but remain largely latent.	**3**—reports consistent improvements vs. strong embedding baselines (including large average F1 gains on multi-interaction triples).	**3**—uses a shallow (single ENC-SCO) architecture and modest training regime for downstream probing.	ChemProt and DrugProt knowledge graphs from BioCreative
[53]	**2**-CETSA PPI prediction using decision trees; not tested on large datasets	**3**—uses decision trees, which are transparent and easy to interpret	**2**—good matching of predicted PPI scores, but lacks extensive benchmarking	**2**—Decision trees are efficient, but iterative clustering adds computational overhead	CETSA, (HCT116, HEK293T) and Bioplex database
[54]	**4**—applies node representation learning to over 19,000 proteins across 23,000+ diseases	**2**—Embedding methods like node2vec are less transparent but biologically validated	**3**—achieved 0.90 recall and 0.79 F1 score in drug target prediction	**3**—Embedding and Naïve Bayes classification ensure fast prediction on large-scale graphs	STRING, TTD, DisGeNET
[55]	**2**—tested on small datasets with up to 493 protein complexes; may not scale well	**2**—Fuzzy logic enhances uncertainty handling but reduces model clarity	**3**—GAFNB outperformed Naïve Bayes, demonstrating robustness to noisy data	Moderate—Genetic algorithm and fuzzy modeling increase computational demands	MIPS and TAP-MS datasets
[56]	**3**—Framework integrates gene expression and PPI data for multiple cancers	**2**—Ensemble models (SVM + NB) reduce explainability	**3**—Achieved 15â€″20% improvement over baseline classifiers on cancer datasets	**3**—Ensemble method balances training time with strong classification performance	Microarray cancer datasets and sequence PPI data
[57]	**3**—ensemble of nine pipelines with stacking, allowing large hotspot prediction	**1**—Complex model with stacking & multiple algorithms limits transparency	**3**—Achieved accuracy of 0.8462 using ensemble voting and stacking	**2**—Stacking ensemble increases computational time despite performance gain	SpotOn and extracted features from HotPoint
[58]	**4**—designed for multi-task learning with flexible pre-training on large complexes	**1**—Deep geometric learning models and equivariant encoding are complex	**3**—outperformed existing models on four benchmark datasets for PPI prediction	**3**—Multi-task pre-training accelerates downstream prediction with good generalization	Four benchmark PPI mutation datasets
[59]	**3**—capable of predicting 411 new dengue-human PPIs using SVM	**3**—Traditional models like SVM, NB, and KNN offer good model transparency	**3**—SVM achieved superior accuracy in dengue-human PPI prediction.	**3**—Supervised learning models ensure efficient processing with cross-validation.	DenvInt database of 692 dengue-human interactions

**Table 4:** Evaluating research papers that have employed Convolutional Neural Networks (CNNs) technique.

	Scalability	Interpretability	Accuracy	Efficiency	Datasets used
[61]	**2**—requires sequence embeddings plus AlphaFold2 structure graphs and GearNet/BAN processing, which is heavy.	**3**—bilinear attention weight maps can be mapped to specific residues to highlight significant interaction sites.	**4**—reported to outperform other state-of-the-art methods across four datasets.	**2**—deep multimodal pipeline increases compute and memory compared to simpler baselines.	Yeast, Yeast_10, Multi-species, Multi-interaction type (STRING).
[62]	**3**—uses 1D CNN + GCN and emphasizes reduced complexity versus attention-heavy models; designed for large multi-label datasets.	**2**—uses explicit nominal physicochemical features (more interpretable than latent-only encodings); lacks residue attention explanations	**3**—consistent Micro-F1 gains (3.81–32.40%) compared with state-of-the-art baselines under BFS/DFS/Random splits.	**3**—nominal features are lower-dimensional and the model avoids heavy attention mechanisms, reducing computation/data needs.	STRING-derived multi-label PPI type datasets; SHS27K, SYS60K
[63]	**2**—multiple attention-based modules across two modalities plus cross-protein fusion increases training/inference cost.	**3**—attention mechanisms can indicate important residues, though interpretation is less than rule-based models	**4** —performance surpassing existing state-of-the-art methods on four benchmark datasets.	**1**—attention and graph modules add overhead compared to lightweight CNN-only approaches.	Yeast, Multi-species, Multi-class, SKEMPI-derived ∆∆G regression.
[64]	**3**—trained on hundreds of thousands of UniProt sequences & large ORF corpora using AE/VAE architectures.	**1**—latent representations and reconstruction error offer limited biological/interaction-level explanations.	**1**—focuses on sequence generation/reconstruction rather than interaction prediction accuracy.	**2**—autoencoder/conv-VAE training is generally efficient, but large-scale data impose compute costs	UniProt protein sequences, plus bacterial ORFs from GTDB genomes.
[65]	**2**—designed for moderate-sized literature datasets with kNN-based filtering;	**3**—based on intuitive metrics like information gain and k-nearest neighbors, which are easy to trace and understand	**3**—outperformed all others in the BioCreative II.5 challenge for document classification tasks	**2**—achieves good performance, but protein mention normalization steps can be time-consuming	BioCreative II.5 ACT: 619 training articles, 599 test articles
[66]	**2**—handles biological literature efficiently but limited by feature complexity and external tools (e.g., NLP pipelines)	**2**—combines SVMs and CRFs with custom feature engineering; interpretable but complex in design	**3**—ranked among the top-performing systems in the BioCreative II.5 challenge on two tasks	**2**—uses NLP tools and multiple ML models, which may introduce overhead	BioCreative II.5 INT and IPT tasks
[67]	**2**—suitable for moderate datasets, but does not scale well to large protein structures	**2**—ensemble learning approach is explainable, but its component models are not	**3**—outperformed several existing techniques in hot spot prediction	**2**—Ensemble SVM system incurs computational cost in feature selection	Protein hot regions and hot spots
[68]	**3**—uses GPU acceleration to make SVMs scalable to high-dimensional & larger datasets	**2**—SVM models with RBF kernels are difficult to interpret.	**3**—demonstrated superior accuracy on five public PPI datasets	**4**—achieves significant training time reduction via GPU acceleration	Five public PPI datasets (not named)
[69]	**2**—suitable for standard PPI datasets but lacks scalability enhancements	**2**—Residue & spatial profiles are useful but require domain knowledge for interpretation	**3**—reported strong recall rates and general performance across dataset types	**3**—ELM implementation achieves faster training compared to SVM	563 non-redundant protein chains from PDB
[70]	**2—**suitable for domain-specific applications such as HIV-1′ Human PPI data	**2**—uses multivariate mutual information features, which are meaningful but complex	**3**—achieved average accuracy between 83.5% and 84.9% across MMI types	**2**—SVM performance is acceptable but not highly optimized	NCBI HIV-1-′ Human PPI dataset
[71]	**3**—uses Mercer series to approximate SVM kernel, reducing computational load	**2**—Mercer series low-rank is complex but improves transparency over black-box models	**3**—matches kernel-based SVM accuracy with reduced computation	**3**—dramatically lowers SVM training time via low-rank approximation	S. Cerevisiae PPI dataset

**Table 5:** Evaluating research papers that have employed Support Vector Machine (SVM) technique.

	Scalability	Interpretability	Accuracy	Efficiency	Datasets Used
[76]	**3**—Ensemble framework scales reasonably to medium–large PPI networks, but feature extraction (65 features) and heuristic search increase computational load.	**2**—Structural modularity is interpretable, but ensemble VotingRegressor reduces transparency of individual decision contributions.	**4**—outperforms 12 state-of-the-art methods; integrates biological + topological features and ensemble learning for robust detection.	**2**—Weighted network construction and core mining are computationally intensive.	Gavin, Krogan core, DIP, MIPS PPI networks; Standard protein complexes 1 & 2
[77]	**2**—Quantum models are promising but limited by quantum hardware scalability and computational resources.	**2**—Quantum kernel space and quantum feature mapping reduce model transparency compared to classical SVM.	**4**—achieves highest AUC (0.923 human; 0.927 C. elegans) outperforming classical SVM, KNN, and RF.	**3**—Quantum parallelism improves kernel efficiency; speed depends on hardware constraints.	Human CPI dataset; C. elegans CPI dataset (1434 compounds)
[78]	**3**—BioLMiner system processed large-scale literature using SVM-models for interaction extraction.	**2**—integrates SVMs and CRFs with hybrid recognition models, reducing transparency	**3**—top-performing systems in the BioCreative II.5 challenge on interaction	**2**—Multi-stage architecture increases complexity but supports interaction	BioCreative II.5 challenge datasets (INT and IPT tasks)
[79]	**2**—focuses on prediction of hot regions using SVM ensemble; dataset is limited to 16 protein complexes	**2**—Ensemble SVM enhances performance but reduces interpretability of individual predictions	**3**—demonstrated superior accuracy over baseline methods in identifying hot regions and residues	**3**—Ensemble learning with mRMR feature selection ensures effective computation despite complexity	ASEdb dataset with 16 protein complexes
[80]	**4**—GPU-accelerated SVM framework supports efficient training on high-dimensional datasets	**2**—Kernel-based SVMs provide strong performance but reduce model transparency	**3**—achieved faster and more accurate hyperparameter estimation	**4**—utilized GPU parallelization for kernel matrix, reducing training time	classification & PPI datasets, including host–pathogen PPI
[81]	**3**—Mercer series-based low-rank kernel approximation enables scalable SVM training on sequence-based PPI data.	**2**—Kernel transformations introduce complexity, but Mercer series approximation aids understanding	**3**—maintained high prediction performance with reduced computational requirements	**3**—significantly reduced SVM training time using Hilbertâ€″Schmidt SVD for kernel approximation	S. cerevisiae protein interaction dataset
[82]	**2**—applied SVM with multivariate mutual information to moderate-sized HIV1-human dataset	**3**—use of interpretable MMI descriptors with SVM supports transparent reasoning.	**2**—up to 84.90% accuracy, but SVM models lag behind ensemble methods	**3**—fast feature extraction and classification with computational resources	NCBI HIV-1 and protein interaction dataset

**Table 6:** Evaluating research papers that have employed Least Squares SVM (LS-SVM) technique.

	Scalability	Interpretability	Accuracy	Efficiency	Datasets Used
[83]	**2**—applied to 17 PP complexes with limited training samples; scalability to large datasets is not demonstrated.	**3**—LS-SVM with Bayesian provides structured learning and interpretable feature selection	**3**—achieved an F1-score of 0.84 in cross-validation and 0.58 in independent testing.	**3**—Bayesian inference eliminates cross-validation overhead, enabling faster hyperparameter tuning.	ASEdb, BID, Cho’s dataset (17 complexes), 158 labeled residues (65 hot spots, 93 non-hot spots)
[84]	**4**—tested on large-scale and cross-species datasets (Yeast, H. pylori, C. elegans), demonstrating excellent scalability.	**2**—uses PCA for dimension reduction and RVM; decision paths are less interpretable.	**4**—achieved accuracy of 92.98% (yeast) and 95.58% (H. pylori), outperforming SVM.	**3**—PCA and RVM significantly reduce feature noise and computational cost compared to traditional classifiers	Yeast, H. pylori, C. elegans, M. musculus, H. sapiens, E. coli (11,188 yeast, 2916 H. pylori)

**Table 7:** Evaluating research papers that have employed Naïve Bayes technique.

	Scalability	Interpretability	Accuracy	Efficiency	Datasets
[85]	**4**—AutoTarget embedded over 19,000 proteins and associated them with over 23,000 diseases, which supports large analysis	**2**—uses node embeddings and Naïve Bayes, which reduces transparency.	**3**—has a recall of 0.90 and an F1 score of 0.79, validated with case studies and clustering.	**3**—Node2vec+ and Naïve Bayes combination ensures scalability and efficiency	STRING, Therapeutic Target
[86]	**2**—applied to two datasets with 493 protein complexes; scalability not demonstrated for very large networks.	**2**—Fuzzy modeling handles uncertainty, but fuzzy feature matrices reduce transparency	**3**—GAFNB outperformed standard Naïve Bayes, effectively handling noisy data.	**2**—Genetic algorithm and fuzzy logic increase computational complexity.	MIPS and TAP-MS datasets
[87]	**3**—classifies cancer across multiple datasets using ensemble learning on sequence and gene expression data.	**2**—SVM and NB ensemble improve prediction but reduce transparency.	**3**—outperformed baseline classifiers with 15–20% improvement across metrics.	**3**—Principal Component Analysis & ensemble methods enable fast classification	Kaggle cancer gene expression
[88]	**3**—utilizes a nine-pipeline ensemble with SMOTE and PCA, capable of handling large feature sets and sample imbalance.	**1**—Stacking and ensemble pipelines involve multiple models, limiting transparency and interpretability.	**3**—achieved high prediction accuracy using ensemble stacking of models like XGBoost	**2**—Ensemble setup provides strong results but requires more computational resources and tuning.	SpotOn; features derived-protr package

**Table 8:** Evaluating research papers that have employed Probabilistic Decision Tree technique.

	Scalability	Interpretability	Accuracy	Efficiency	Datasets
[25]	**3**—integrated large-scale PPI and miRNA-target networks using BioGRID and HPRD datasets.	**2**—Gradient Boosting offers limited interpretability, but enhances understanding.	**3**—achieved F1-score of 0.82 with (0.91) and recall (0.77).	**3**—SMOTE balancing with GBC, with manageable computational cost.	BioGRID, HPRD (9455 genes)
[89]	**3**—applied to yeast datasets with tens of thousands of interactions, showing good scalability in PPI detection	**2**—boosted decision trees offer some transparency; use of topological features aids interpretability.	**4**—precision of 0.994 and recall of 1.0 in predicting 6531 interactions, with 22/37 links validated	**3**—used a 20-tree ensemble with efficient feature extraction; handled large datasets effectively.	Yeast PPI networks (Collins; STRING)
[90]	**2**—focused on two cell lines and a limited number of protein pairs; scalability not demonstrated beyond this scope.	**3**—Decision tree model and iterative clustering provide clear and interpretable results.	**3**—MAE of 0.0698 and matching histograms show close alignment with ground truth.	**2**—Multiple validation folds and clustering increase runtime despite good accuracy.	HCT116, HEK293T; and Bioplex PPI

**Table 9:** Evaluating research papers that have employed KNN.

	Scalability	Interpretability	Accuracy	Efficiency	Datasets Used
[91]	**3**—designed to scale across multiple mutation datasets with varying sizes and mutation types.	**2**—uses graph neural networks and multi-task learning, which offer limited transparency.	**3**—outperforms baseline models across all tested datasets in terms of Pearson’s correlation coefficient.	**2**—uses GBT decoders and geometric encoders; efficiency depends on pretraining and mutant generation method.	AB-Bind (S645, M1101), SKEMPI (S1131), SKEMPI2 (S4169)
[92]	**3**—applies to large host–pathogen datasets using sequence-derived features.	**3**—KNN, SVM, and Naïve Bayes with understandable feature spaces (AAC, conjoint triad).	**2**—SVM performs best, but KNN provides useful comparisons; accuracy varies.	**3**—Simple sequence-based features ensure fast training and inference.	DenvInt, HPRD (negative set), human proteins
[93]	**4**—over 5000 organisms and 24.6 million proteins with large-scale integration.	**2**—aggregates from multiple channels and algorithms; network-level interpretations are complex	**3**—combines curated and predicted interactions with benchmarked confidence scores.	**3**—optimized for fast access and large-network visualization; web/API and Cytoscape tools	STRING database (experimental, predicted)
[94]	**2**—applies to document sets, but depends on cross-validation costs.	**3**—clear feature representation and distance-based similarity in document classification	**3**—Outperformed other BioCreative II.5 submissions in precision-recall AUC.	**2**—Custom IG-weighted KNN and normalization steps increase computation time.	BioCreative II.5 corpus (FEBS publications)

**Table 10:** Evaluating papers that employed Weighted K-Nearest Neighbor (WKNN) technique.

	Scalability	Interpretability	Accuracy	Efficiency	Datasets Used
[95]	**3**—designed for large-scale functional annotation across whole proteomes; scalable with batch processing and integration into annotation pipelines.	**2**—The system’s layered structure and regression-based scoring offer interpretability, but statistical modeling and score combinations add complexity.	**3**—outperformed competing tools in free-text and GO annotation tasks, and showed superior results in CAFA evaluations and benchmarks like NOSELF	**3**—optimized sequence filtering, clustering, and sparse regression models reduce redundant processing and improve annotation speed.	UniProtKB, SwissProt datasets (for training/evaluation); NOSELF and NOCLOUD filtered versions for benchmarking.
[96]	**2**—handles multiple GEO datasets but is limited to predefined morphine-related studies; less suitable for continuous or large-scale data streams.	**3**—uses WKNN clustering and network-based visualization (mRNA–miRNA–PPI), which enhances biological interpretation and clarity of molecular interactions.	**3**—identified common DEGs for addiction and analgesia; robust clustering and pathway enrichment (e.g., GO/KEGG); identified miR-129 and Fos as candidate markers.	**2**—PCA, DEG screening, and enrichment analyses are computationally standard; integration of multiple networks is more resource-intensive.	GEO datasets: GSE62346, GSE50382, GSE9525, GSE7762, GSE78280, GSE15774 (covering both analgesic and addiction effects).

**Table 11:** Comparative Quantitative Analysis of all techniques.

Technique	Avg. Accuracy (%)	Avg. F1-Score (%)	Computational Time	Notes
**Graph Neural Networks**	97	96	**Moderate**	High accuracy; suitable for large-scale datasets; moderate computational time.
**Deep Neural Networks**	96	92	**High**	High accuracy; computationally intensive.
**Convolutional Neural Networks**	93	89	**High**	Accuracy varies with dataset; computationally intensive.
**Extreme Learning Machine**	94	92	**Low**	Fast training; good accuracy; efficient for large datasets.
**Support Vector Machine**	91	86	**Moderate**	Accuracy depends on kernel and features; moderate computation time
**Least Squares SVM**	93	91	**Moderate**	Comparable accuracy to SVM; computational time varies with implementation.
**K-Nearest Neighbor**	90	91	**High**	Simple implementation; computationally intensive during inference.
**Weighted K-Nearest Neighbor**	92	92	**High**	Similar to KNN with weighting; computationally intensive.
**Naïve Bayes**	81	75	**Very Low**	Fast and simple; lower accuracy compared to other methods.
**Probabilistic Decision Tree**	88	86	**Moderate**	Balanced accuracy and computational time.

**Table 12:** A comprehensive observational analysis of the Supervised Learning techniques for PPI detection covered in the survey.

	Dataset Suitability	Scalability	Interpretability	Efficiency	Strengths	Limitations
**Naïve Bayes**	Best for small to moderately sized datasets—handles well with simple, less correlated features.	***Highly scalable***—computational complexity grows linearly with data size.	***High***—provides probabilistic outputs that are easy to interpret.	***Extremely efficient***—requires minimal computation for both training and prediction.	Fast training, interpretable model, suitable for early-stage analysis and filtering.	Assumes independence between features, which is unrealistic for biological data and lowers accuracy.
**KNN**	Suitable for small to moderate datasets—performance drops with large datasets due to memory and time constraints.	***Poor scalability***—requires full dataset scan at inference time, increasing with dataset size.	***High***—prediction is based on simple distance comparison; easy to explain.	***Low***—high computational cost during inference phase due to distance calculations.	Simple implementation with no training required; supports diverse distance metrics	Inefficient on large datasets; sensitive to noise and irrelevant features.
**WKNN**	Best for moderate datasets where small distance variations carry biological meaning.	***Poor scalability***—still requires evaluating all training samples during inference.	***Moderate to High***—provides interpretability through weighted influence of neighbors	***Low***—adds complexity over KNN by applying distance-based weighting.	Enhances KNN by emphasizing closer, relevant neighbors; improves prediction a	Suffers from the same scalability issues as KNN; performance degrades on large-scale datasets.
**SVM**	Effective for moderate, high-dimensional datasets—particularly binary classification tasks.	***Limited scalability***—training has quadratic time complexity; may struggle with large data	***Low***—complex kernel decisions are hard to interpret.	***Moderate***—depends on kernel and number of support vectors.	High accuracy; robust generalization in high-dimensional and non-linear spaces.	Requires hyperparameter tuning and kernel selection; performance bottlenecks in very large dataset
**LS-SVM**	Suitable for structured, high-dimensional data—performs well when features are optimized.	***Moderate***—linear equation-based formulation helps with moderate-sized datasets.	***Moderate***—Bayesian variants allow some interpretability, though not as intuitive as trees	*Moderate to High*—training is efficient due to least squares formulation.	Achieves accuracy similar to SVM with reduced computational burden and fast convergence.	Trade-off between transparency and speed; may not outperform deep models for complex features
**ELM**	Best for real-time, large-scale applications—fast training makes it suitable for big datasets.	***Limited scalability***—fixed random weights can reduce generalization if not tuned.	***Low***—less transparent due to randomly assigned hidden layer weights.	***Very High***—avoids iterative weight updates; uses analytical solution for output layer.	Extremely fast model training and inference; ideal for time-sensitive & streaming data tasks	Less flexible than backpropagation-based models; vulnerable to noise and variance in input data
**DNNs**	Suitable for large, complex datasets—excels in discovering non-linear and hierarchical patterns.	***High***—highly scalable with GPU acceleration and parallelization.	***Low***—multi-layered black-box models hinder explainability.	***Low to Moderate***—requires long training times and substantial computation resources.	Learns intricate protein interaction representations; supports feature learning	Prone to overfitting on small datasets; needs large labeled data and hyperparameter tuning.
**CNNs**	Best for sequence-based and spatial data—captures motifs and local dependencies in proteins.	***Moderate to High***—pooling layers reduce data size; GPU-friendly design.	***Low to Moderate***—convolutional layers are harder to interpret than decision trees.	***Low to Moderate***—heavy training phase but manageable inference time with optimized hardware.	Automatically learns spatial features from protein sequences; avoids manual feature engineering.	Needs high-quality, well-structured inputs; training can be resource-intensive.
**GNNs**	Ideal for graph-structured protein data—models both local and global interaction patterns.	***High***—capable of scaling to large interaction networks using message-passing architectures	***Moderate***—some explainability via node feature tracing, though still complex	***Moderate***—graph construction and training can be demanding but manageable	Captures structural and functional relationships; well-suited for complex networks.	Requires prior graph knowledge or construction; complex implementation and tuning.
**Probabilistic Decision Tree**	Effective for moderately sized datasets with uncertainty in labels—handles noise well.	***Moderate***—scalability depends on tree depth and dataset structure.	***High***—decision paths are transparent and easily explainable.	***Moderate***—model training and prediction are reasonably fast.	Probabilistic outputs offer soft classifications; interpretable and intuitive.	Can overfit; not ideal for complex dependencies.

**Table 13:** Dataset size and Specific Recommendations.

**Dataset Size**	**Recommended Methods**
**Small**	Naïve Bayes (simplicity), SVM (accuracy), WKNN (accuracy with small sets)
**Moderate**	ELM (fast and accurate), CNN (spatial patterns), LS-SVM (robust with moderate cost)
**Large**	GNN (scalability with relational data), DNN (deep abstraction), ELM (real-time use)
**Use Case Recommendations**	
**Use Case**	**Best Techniques**
**High Interpretability**	Probabilistic Decision Tree, Naïve Bayes
**Accuracy-Critical Tasks**	GNN, DNN, LS-SVM, SVM
**Real-Time Applications**	ELM, Naïve Bayes
**Topological Dependencies**	GNN
**Protein Sequence Modeling**	CNN, DNN

**Table 15:** Scores of the algorithms in terms of **Sensitivity**. The figures also include rankings for the various techniques.

Technique	Selected Papers	Datasets	Score	Technique Rank
**Graph Neural Network (GNN)**	[30]	DIP	90.23	1
		HPRD	89.47	
		STRG	88.65	
**Convolutional Neural Network**	[66]	DIP	88.71	3
		HPRD	87.89	
		STRG	86.95	
**Extreme Learning Machine (ELM)**	[72]	DIP	86.16	7
		HPRD	85.56	
		STRG	84.93	
**Support Vector Machine (SVM)**	[80]	DIP	85.78	9
		HPRD	85.13	
		STRG	84.14	
**Least Squares SVM (LS-SVM)**	[83]	DIP	87.61	5
		HPRD	86.94	
		STRG	86.01	
**Probabilistic Decision Tree**	[89]	DIP	88.10	4
		HPRD	87.57	
		STRG	86.32	
**K-Nearest Neighbor (KNN)**	[94]	DIP	85.17	10
		HPRD	84.33	
		STRG	83.52	
**Weighted KNN (WKNN)**	[95]	DIP	86.43	8
		HPRD	85.51	
		STRG	84.65	
**Deep Neural Network (DNN)**	[55]	DIP	89.34	2
		HPRD	88.71	
		STRG	87.82	
**Naïve Bayes-Based**	[85]	DIP	87.52	6
		HPRD	86.43	
		STRG	85.44	

**Table 16:** Scores of the algorithms in terms of **Specificity**. The figures also include rankings for the various techniques.

Technique	Selected Papers	Datasets	Score	Technique Rank
**Graph Neural Network (GNN)**	[30]	DIP	89.12	1
		HPRD	88.33	
		STRG	86.77	
**Convolutional Neural Network (CNN)**	[66]	DIP	87.44	3
		HPRD	86.22	
		STRG	84.91	
**Extreme Learning Machine (ELM)**	[72]	DIP	82.84	10
		HPRD	82.26	
		STRG	80.89	
**Support Vector Machine (SVM)**	[80]	DIP	84.32	8
		HPRD	83.08	
		STRG	81.84	
**Least Squares SVM (LS-SVM)**	[83]	DIP	85.23	6
		HPRD	84.14	
		STRG	82.48	
**Probabilistic Decision Tree**	[89]	DIP	85.78	5
		HPRD	85.16	
		STRG	82.60	
**K-Nearest Neighbor (KNN)**	[94]	DIP	83.95	9
		HPRD	82.43	
		STRG	81.38	
**Weighted KNN (WKNN)**	[95]	DIP	84.74	7
		HPRD	83.11	
		STRG	82.06	
**Deep Neural Network (DNN)**	[55]	DIP	88.01	2
		HPRD	87.10	
		STRG	85.45	
**Naïve Bayes-Based**	[85]	DIP	87.20	4
		HPRD	86.61	
		STRG	83.04	

**Table 17:** Scores of the algorithms in terms of **Precision**. The figures also include rankings for the various techniques.

Technique	Selected Papers	Datasets	Score	Technique Rank
**Graph Neural Network (GNN)**	[30]	DIP	86.81	1
		HPRD	85.74	
		STRG	84.33	
**Convolutional Neural Network (CNN)**	[66]	DIP	85.43	3
		HPRD	84.16	
		STRG	82.92	
**Extreme Learning Machine (ELM)**	[72]	DIP	82.72	7
		HPRD	82.07	
		STRG	81.37	
**Support Vector Machine (SVM)**	[80]	DIP	82.85	9
		HPRD	82.23	
		STRG	80.46	
**Least Squares SVM (LS-SVM)**	[83]	DIP	83.64	6
		HPRD	82.98	
		STRG	81.29	
**Probabilistic Decision Tree**	[89]	DIP	83.78	5
		HPRD	82.55	
		STRG	80.96	
**K-Nearest Neighbor (KNN)**	[94]	DIP	82.57	10
		HPRD	81.35	
		STRG	79.73	
**Weighted KNN (WKNN)**	[95]	DIP	83.01	8
		HPRD	81.97	
		STRG	80.82	
**Deep Neural Network (DNN)**	[55]	DIP	85.96	2
		HPRD	84.89	
		STRG	83.48	
**Naïve Bayes-Based**	[85]	DIP	84.24	4
		HPRD	83.12	
		STRG	81.72	

**Table 18:** Summary of the experimental findings for different algorithms and techniques, and their corresponding literature correlations.

	Summary of Findings	Literature Correlation
**GNN [30]**	**Top performer across all three metrics**: GNN achieved the highest scores in sensitivity. This aligns with the literature that reports GNNs’ superior ability to capture complex topological dependencies in PPI networks by leveraging graph structure and message passing. Their capacity to model both local and global protein interactions reduces false negatives and false positives, contributing to balanced and strong results across all metrics.	Zitnik and Leskovec [99] demonstrated that GNNs can successfully integrate heterogeneous topological information to infer functional protein associations. The ability to propagate context-aware signals across network layers aligns with GNN’s superior performance in modeling PPI relationships.
**DNN [55]**	**Second-best overall**: DNN consistently ranked second in sensitivity, specificity, and precision. DNNs excel at learning non-linear relationships and rich hierarchical representations from large-scale sequence data. The high precision indicates confident predictions with fewer false positives, and the strong sensitivity suggests robust detection of true interactions, making DNNs ideal for complex bioinformatics datasets.	Wang et al. [100] showed that deeper layers significantly improve the model’s capacity to detect complex structural and sequential dependencies. Their findings underscore DNNs’ strength in capturing rich and abstract features necessary for accurate interaction modeling in large datasets.
**CNN [66]**	**High performance with emphasis on sensitivity and precision**: CNN scored well in sensitivity and precision, ranking third, and achieved strong specificity. These results are consistent with CNN’s capacity to learn local sequence motifs and spatial patterns important for interaction prediction. CNNs are often preferred when the feature space is based on protein sequences or structural motifs.	Li et al. [101] demonstrated the use of CNNs for learning sequence motifs in enzyme classification. The convolutional layers effectively extracted spatial features that represent biological patterns. This corroborates CNN’s applicability to PPI tasks, where local sequence or structural features play a critical role in determining interaction likelihood.
**Naïve Bayes [85]**	**Surprisingly strong in specificity and precision**: NB demonstrated competitive specificity and precision, but slightly lower sensitivity. Its assumption of feature independence simplifies modeling and often performs well in high-dimensional but sparse biological datasets. While not as expressive as DNNs or GNNs, it is computationally efficient and still competitive when data preprocessing and feature engineering are well-optimized.	Domingos and Pazzani [102] emphasized Naïve Bayes’ efficiency but also its limitations for tasks requiring sophisticated relationships between features
**Prob. Decision Tree [89]**	**Well-balanced performance**: The technique delivered solid sensitivity, specificity, and precision. Its strength lies in its interpretability and flexibility, particularly when modeling uncertain biological data. The literature indicates that probabilistic trees manage data noise and uncertainty better than standard decision trees, explaining their respectable performance.	Murthy [103] discusses its effectiveness for simpler problems but acknowledges limitations for high-dimensional data.
**LS-SVM [83]**	**Moderate across the board**: LS-SVM showed better sensitivity and precision compared to standard SVM, with decent specificity. The least squares formulation simplifies the optimization problem, reducing computational cost while preserving performance. It is more stable in noisy datasets, as often encountered in PPI prediction.	[104] Suykens and Vandewalle [4k] emphasized LS-SVM’s applicability to classification problems with noisy or high-dimensional data—a context fitting PPI datasets where standard SVM may underperform.
**WKNN [95]**	**Incremental improvements over KNN**: WKNN outperformed KNN in sensitivity (86.43 vs. 85.17), specificity (84.74 vs. 83.95), and precision (83.01 vs. 82.57), reflecting its advantage in using distance-weighted voting. This aligns with the literature that shows WKNN’s ability to reduce the influence of noisy or distant neighbors, leading to better precision and general performance in high-dimensional biological datasets.	Chen et al. [105] incorporated a distance-weighted voting scheme into KNN. Their integrative approach reduced the impact of misleading neighbors by assigning higher influence to closer instances, which significantly improved prediction precision. This finding supports WKNN’s effectiveness in handling complexity in PPI datasets
**ELM [32]**	**Moderate sensitivity and precision, but lowest specificity**: ELM had decent sensitivity (86.16) and precision (82.72), but its specificity (82.84) lagged behind other neural methods. Although ELM is known for fast training due to random hidden weights and analytical output layer computation, this architecture may generalize less effectively on complex data, which explains the relatively higher false positive rate.	Huang et al. [106] showed that while ELM is fast, it lacks the adaptability of more advanced methods for intricate data like PPI detection
**SVM [32]**	**Lower-tier performance in this comparison**: SVM had lower sensitivity, specificity, and precision compared to LS-SVM and modern neural methods. Though historically strong in bioinformatics, SVM’s limitations on large, complex, and noisy datasets reduce its competitiveness when compared to more flexible deep learning models. Kernel choice and parameter tuning significantly impact its performance.	Cortes and Vapnik [107] demonstrated SVM’s robustness in classification tasks, but modern ensemble methods often surpass it in large-scale datasets
**KNN [32]**	**Lowest performing method overall**: KNN ranked lowest in sensitivity, specificity, and precision. The literature attributes this to its poor scalability and high sensitivity to irrelevant features and noise—especially problematic in PPI datasets with high dimensionality and heterogeneity. Despite its simplicity and interpretability, its performance does not scale well without enhancements such as feature selection or dimensionality reduction.	Cover and Hart [108] identified KNN’s simplicity but noted its shortcomings in noisy and high-dimensional environments.

## Data Availability

No new data were created or analyzed in this study. Data sharing is not applicable to this article.
